# Changes in transcription of cytokinin metabolism and signalling genes in grape (*Vitis vinifera* L.) berries are associated with the ripening-related increase in isopentenyladenine

**DOI:** 10.1186/s12870-015-0611-5

**Published:** 2015-09-16

**Authors:** Christine Böttcher, Crista A. Burbidge, Paul K. Boss, Christopher Davies

**Affiliations:** CSIRO Agriculture Flagship, Waite Campus, WIC West Building, PMB2, Glen Osmond, South Australia 5064 Australia

**Keywords:** Cytokinins, Isopentenyladenine, *Vitis vinifera*, Ripening

## Abstract

**Background:**

Cytokinins are known to play an important role in fruit set and early fruit growth, but their involvement in later stages of fruit development is less well understood. Recent reports of greatly increased cytokinin concentrations in the flesh of ripening kiwifruit (*Actinidia deliciosa* (A. Chev.) C.F. Liang & A.R. Ferguson) and grapes (*Vitis vinifera* L.) have suggested that these hormones are implicated in the control of ripening-related processes.

**Results:**

A similar pattern of isopentenyladenine (iP) accumulation was observed in the ripening fruit of several grapevine cultivars, strawberry (*Fragaria ananassa* Duch.) and tomato (*Solanum lycopersicum* Mill.), suggesting a common, ripening-related role for this cytokinin. Significant differences in maximal iP concentrations between grapevine cultivars and between fruit species might reflect varying degrees of relevance or functional adaptations of this hormone in the ripening process. Grapevine orthologues of five Arabidopsis (*Arabidopsis thaliana* L.) gene families involved in cytokinin metabolism and signalling were identified and analysed for their expression in developing grape berries and a range of other grapevine tissues. Members of each gene family were characterised by distinct expression profiles during berry development and in different grapevine organs, suggesting a complex regulation of cellular cytokinin activities throughout the plant. The post-veraison-specific expression of a set of biosynthesis, activation, perception and signalling genes together with a lack of expression of degradation-related genes during the ripening phase were indicative of a local control of berry iP concentrations leading to the observed accumulation of iP in ripening grapes.

**Conclusions:**

The transcriptional analysis of grapevine genes involved in cytokinin production, degradation and response has provided a possible explanation for the ripening-associated accumulation of iP in grapes and other fruit. The pre- and post-veraison-specific expression of different members from each of five gene families suggests a highly complex and finely-tuned regulation of cytokinin concentrations and response to different cytokinin species at particular stages of fruit development. The same complexity and specialisation is also reflected in the distinct expression profiles of cytokinin-related genes in other grapevine organs.

**Electronic supplementary material:**

The online version of this article (doi:10.1186/s12870-015-0611-5) contains supplementary material, which is available to authorized users.

## Background

Naturally occurring cytokinins are adenine derivatives whose diverse functions in plant growth and development have earned them recognition as molecules of great biological and agricultural importance. The four most abundant cytokinins found in plants, *trans*-zeatin (*t*Z), *N*^*6*^-(Δ^2^-isopentenyl)-adenine (iP), *cis*-zeatin (*c*Z), and dihydrozeatin, differ in the stereo-isomeric position, hydroxylation and saturation of the isoprenoid side chain [1], but little is known about the physiological relevance of these side chain differences [2]. Apart from their well-described role in regulating cell division and differentiation [3], cytokinins are involved in a range of processes essential to plant survival, such as leaf senescence [4, 5], control of shoot-to-root balance [6, 7], nutritional signalling [8, 9], stress tolerance [10] and nodulation [11, 12]. Quantity and composition of cellular cytokinins are regulated through biosynthesis, transport, inter-conversion of distinct forms, transient inactivation by conjugation, and irreversible inactivation by side chain cleavage [13]. The targeted disturbance of this balance, leading to increased activity of inflorescence and floral meristems and higher seed yield in rice (*Oryza sativa* L.) [14] and Arabidopsis (*Arabidopsis thaliana* L.) [15], has recently provided evidence for the importance of cytokinins in reproductive development and hence crop productivity. In support of this, high cytokinin activities or concentrations have been reported in immature seeds and fruit from a large number of species, including pea (*Pisum sativum* L.) [16], white lupine (*Lupinus albus* L.) [17], Christmas rose (*Helleborus niger* L.) [18], tomato (*Solanum lycopersicum* Mill.) [19], strawberry (*Fragaria ananassa* Duch.) [20], kiwifruit (*Actinidia deliciosa* (A. Chev.) C.F. Liang & A.R. Ferguson) [21], raspberry [22] and grape (*Vitis vinifera* L.) [23–25]. Generally, cytokinin activities/concentrations were found to peak shortly after fertilization coinciding with periods of high rates of cell division, which has linked these hormones to fruit set and early fruit growth [26, 27]. Applications of synthetic cytokinins such as 6-benzylaminopurine, N-(2-Chloro-4-pyridinyl)-N’-phenylurea (CPPU) and thidiazuron (TDZ) have been widely used in fruit such as grape [28], kiwifruit [29], blueberry (*Vaccinium ashei* Reade) [30], apple (*Malus domestica* Borkh.) [31] and pear (*Pyrus communis* L.) [32] to improve fruit set and/or increase fruit size. In contrast, the role of cytokinins during later stages of fruit development is less well documented and understood, partly due to the often reported decrease in cytokinin activities/concentrations following the initial growth phase [33]. Treatment of fruit with the above mentioned cytokinins has produced inconsistent effects on the progression of ripening varying with fruit species and cytokinin used. For example, CPPU-treated grapes showed a delayed accumulation of sugars and anthocyanins and remained firmer than control berries [34] and a similar CPPU-induced ripening delay has been described in blueberry [30]. However, the opposite effect was observed in kiwifruit, where CPPU treatment led to increased sugar accumulation, decreased acidity and reduced flesh firmness [35]. TDZ had the same ripening-advancing effect on kiwifruit as CPPU [35], whereas ripening of TDZ-treated persimmon (*Diospyros kaki* L.) fruit was delayed, as evidenced by a delay in sugar accumulation and chlorophyll degradation [36]. In contrast, treatment with 6-benzylaminopurine had no effect on the ripening progression of persimmon [36]. While application studies have therefore not given any clear indications for possible functions of endogenous cytokinins in the ripening process, the asynchronous ripening of siliques and reduced production of viable seeds in cytokinin-deficient Arabidopsis mutants suggest an involvement of these hormones in fruit maturation [6]. In addition, two recent studies on kiwifruit [37] and grape berries [38] have reported a sharp increase in the concentration of active cytokinins in the flesh of ripening fruit. In the case of kiwifruit, the main contributor to this increase was *t*Z, whereas iP was found to be the main cytokinin species accumulating in ripening grapes.

The aim of this study was to further investigate the ripening-related increase in iP concentrations in grapes, focusing on the role of local cytokinin biosynthesis, activation, perception, signalling and degradation. The expression profiles of relevant genes in developing grape berries were indicative of distinct sets of cytokinin-related genes controlling the quantity and composition of, and responsiveness to, cytokinin species accumulating in the fruit during different stages of development. In addition, evidence is provided that the accumulation of iP during the ripening phase is common to a range of grapevine cultivars and also occurs in tomato and strawberry.

## Methods

### Plant material

For the analysis of developmental changes in the expression of cytokinin-related genes and cytokinin levels, *Vitis vinifera* L. cv. Shiraz berries from a commercial vineyard were collected at weekly intervals as described by Böttcher et al. [39] in the 2010/2011 season. All tissues used for gene expression studies in various grapevine organs were collected from Shiraz plants grown in an experimental vineyard or glasshouse in Adelaide, South Australia [39]. In addition to the Shiraz berry series, cytokinin measurements were also taken from the following samples: 1) *Vitis vinifera* L. cv. Cabernet Sauvignon and cv. Riesling, grown at a commercial vineyard (Waikerie, South Australia; −34.100°, 139.842°) and sampled every two weeks as described by Kalua and Boss [40, 41]. Seeds were removed from frozen berries prior to grinding and cytokinin extraction. 2) *Vitis vinifera* L. cv. Pinot Noir berries, grown at a commercial vineyard (Willunga, South Australia; −35.263°, 138.553°) and sampled as in 1), but retaining the seeds. 3) Grapes of similar sugar content (19.4–20.8°Brix) collected from 13 grapevine species (11 *Vitis vinifera*, one *Vitis* hybrid and one interspecific hybrid) grown at an experimental vineyard (Waite Coombe vineyard, Adelaide, South Australia; −34.263°, 138.553°) in the 2013/2014 season. Juice from individual berries (10 berries per replicate, three replicates) sampled from six bunches across two vines was tested for total soluble solids using a PAL-1 digital refractometer (Atago, Tokyo, Japan), followed by immediate deseeding and freezing in liquid nitrogen of berries within the above specified sugar content range. 4) Tomatoes (*Solanum lycopersicum* Mill. var. Moneymaker) grown from seed in the glasshouse (CSIRO Agriculture, Adelaide, South Australia) and harvested at five standard ripening stages as detailed by Böttcher et al. [42]. 5) Strawberries (*Fragaria ananassa* Duch. cv. Ablion) at four different ripening stages (small green, large green, turning, red ripe), sampled at a commercial strawberry farm (Hahndorf, South Australia; −35.038°, 138.816°) in November 2009. A minimum of five strawberries per stage was used for each biological replicate. For a second set of samples, achenes were removed with tweezers prior to freezing in liquid nitrogen.

### Determination of total soluble solids (TSS) levels

Measurements of TSS (degrees Brix) for the berries from the developmental series were done as described by Davies et al. [43].

### Phylogenetic analysis

Grapevine sequences belonging to five families of proteins involved in the biosynthesis, activation, perception, signalling and degradation of cytokinins were identified by BLASTP searches of the non-redundant NCBI protein database (http://www.ncbi.nlm.nih.gov/) using the respective Arabidopsis sequences (see Additional file 1), obtained from The Arabidopsis Information Resource (TAIR; https://www.arabidopsis.org/), as queries. Phylogenetic analyses were conducted using the corresponding nucleotide sequences in MEGA6.06 [44] as follows: The Arabidopsis and grapevine nucleotide sequences for each gene family were aligned using MUSCLE [45], all positions containing gaps and missing data were eliminated. The evolutionary history was inferred by using the Maximum Likelihood method based on the JTT matrix-based model [46]. A bootstrap consensus tree was generated from 100 replicates [47] and branches corresponding to partitions replicated in less than 70 % replicates were collapsed. Initial tree(s) for the heuristic search were obtained automatically by applying Neighbor-Join and BioNJ algorithms to a matrix of pairwise distances estimated using a JTT model and then selecting the topology with superior log value. The coding data was translated assuming a standard genetic code table. The naming of grapevine genes followed the guidelines published by Grimplet et al. [48].

### RNA extraction, cDNA synthesis and qRT-PCR

RNA extraction, cDNA synthesis and qRT-PCR were performed as described previously [49] with modifications as described by Böttcher et al. [39]. The gene-specific primers and corresponding accession number used for *ACT2* (reference gene) have been published previously [50]. All primer pairs for cytokinin-related genes used in this study are listed with corresponding amplicon sizes in Additional file 2. Gene expression data was analysed using the MeV software (version 4.9; http://www.tigr.org/software/tm4/mev.html) and presented as heat maps with hierarchical clustering.

### Extraction and quantification of nucleobase cytokinins

For the quantification of iP and *t*Z, 100 mg of fruit tissue was extracted in 1 mL of 70 % (v/v) ethanol, 0.2 mM diethyldithiocarbamic acid, spiked with 5 pmol of d6-iP and d5-*t*Z (OlChemIm Ltd., Olomouc, Czech Republic) as internal standards, for 2 h at 4 °C on a rotating mixer. After the tissue was pelleted by centrifugation at 4 °C, the supernatant was removed and kept at 4 °C, while the pellet was re-extracted in 1 mL of 70 % (v/v) ethanol, 0.2 mM diethyldithiocarbamic acid for 1 h at 4 °C. Following centrifugation the supernatant was combined with the initial extract, the organic solvent was removed *in vacuo* and the aqueous phase was adjusted to pH 7.5 (NaOH) and applied to a 100 mg C18 SPE column (Waters, Wexford, Ireland). The column was washed with water pH 7.5 (2 mL) and then eluted with 80 % (v/v) MeOH, 2 % (v/v) acetic acid (2.5 mL). The dried residue was re-suspended in 50 μL 90 % (v/v) 15 mM formic acid, adjusted to pH 4.0 with ammonia, 10 % (v/v) methanol to be analyzed with an Agilent LC-MS system (1200 series HPLC coupled with a 6410 triple quad mass spectrometer). The sample (10 μL) was first separated on a Luna C18 column (75 × 4.6 mm, 5 μm, (Phenomenex, Torrance, CA)) held at 30 °C using the following solvent conditions: 0–20 min, linear gradient from 10 % (v/v) MeOH, 90 % 15 mM formic acid, adjusted to pH 4.0 with ammonia to 95 % (v/v) MeOH, 5 % (v/v) 15 mM formic acid, adjusted to pH 4.0 with ammonia, held for 5 min, linear gradient from 95 % (v/v) to 10 % (v/v) MeOH in 1 min, held for 6 min, 0.4 mL min^−1^. The effluent was introduced into the ESI ion source (nebulizer pressure 35 psi) with a desolvation gas temperature of 300 °C at a flow of 8 L min^−1^, with the capillary voltage set to 4 kV. The detection was performed by multiple reaction monitoring in positive ion mode. The optimization of fragmentation was done with iP, *t*Z (Sigma-Aldrich, St. Louis, MO, USA) as well as the labelled standards using the Agilent MassHunter Optimizer software (version B03.01). The following main transitions were used for quantitation: d6-iP 210 > 137, iP 204 > 136, d5-*t*Z 225 > 137, *t*Z 220 > 136. In addition, a qualifier ion transition was included for each compound: d6-iP 210 > 148, iP 204 > 148, d5-*t*Z 225 > 119, *t*Z 220 > 119. The sensitivity of the analysis was enhanced by monitoring d5-*t*Z and *t*Z in a different retention window (0–15 min) to d6-iP and iP (15–22 min). The concentrations of iP and *t*Z in the extracts were quantified in relation to their internal standards using calibration curves that had been generated as follows: 50 μM stocks were used to prepare eight standard solutions (1 nM–500 nM) and 50 μL of each standard solution was mixed with 5 pmol of d6-iP and d5-*t*Z (in triplicate). Samples were dried *in vacuo* and resuspended in 50 μL of 90 % (v/v) 15 mM formic acid, adjusted to pH 4.0 with ammonia, 10 % (v/v) methanol resulting in internal standard concentrations of 100 nM each. A 10 μl-aliquot of each sample was subjected to an LC-ESI-MS/MS analysis as described above and calibration curves were generated using the Agilent Quantification software (version B04.00) by plotting the known concentration of each unlabelled compound against the ratio of analyte peak area to corresponding internal standard peak area. The limits of detection (signal-to-noise ratio >3) gained from the calibration curves were 0.2 fmol μL^−1^ for *t*Z and 0.08 fmol μL^−1^ for iP, the limits of quantification (signal-to-noise ratio >10) were 0.67 fmol μL^−1^ for *t*Z and 0.25 fmol μL^−1^ for iP.

### Statistical data analysis

Significant differences in TSS contents and cytokinin concentrations were identified by analysis of variance (ANOVA) followed by Duncan’s post hoc test. ANOVA was also performed for the gene expression data collected from the Shiraz berry development samples and this was followed by Fisher’s Least Significant Difference (LSD) post hoc test to test for significant differences. Statistical testing of the various datasets was conducted using IBM SPSS Statistics ver. 20 (IBM Australia, Sydney, NSW, Australia).

## Results

### Grape cultivars exhibit similar patterns of cytokinin accumulation during fruit development but iP concentrations at full ripeness vary

**Fig. 1:**
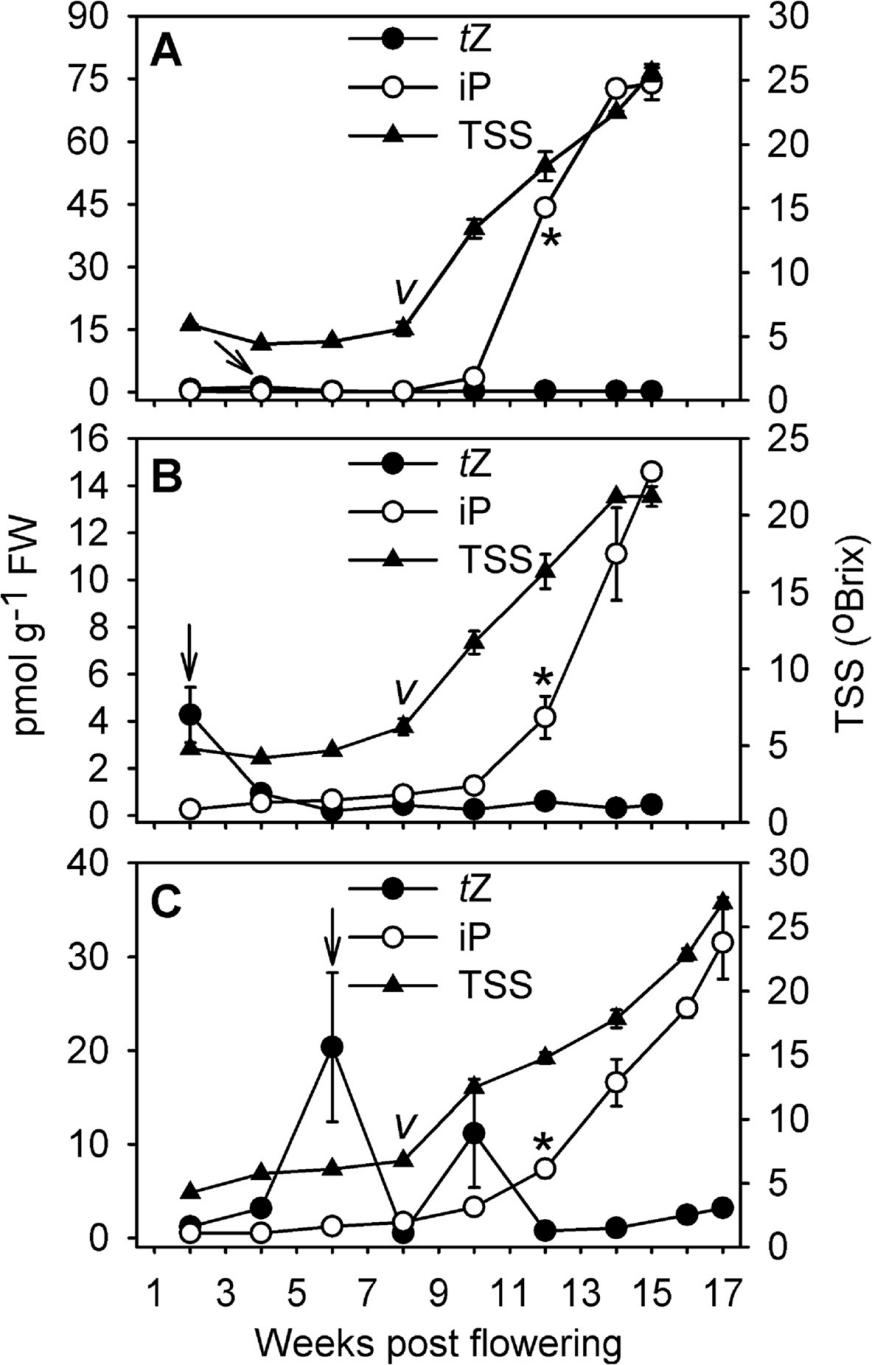
Concentrations of iP and *t*Z in developing berries from three grapevine cultivars.iP and *t*Z were quantified by LC-MS/MS in developing berries of field-grown (**a**) Cabernet Sauvignon, **b** Riesling and **c** Pinot Noir. All data represent means (*n* = 3) ± SE. “*v*” indicates veraison, as determined by the last time point before a significant increase (*p* <0.05) in TSS levels was recorded. Asterisks mark the start of a significant increase in iP concentrations. In each cultivar, the concentration of *t*Z was significantly higher (*p* <0.05) at one time point compared to the others, and this is denoted by an arrow. FW, fresh weight

The recent discovery of a large increase in iP concentrations in ripening Shiraz berries has provided the first evidence for a possible involvement of a cytokinin in the ripening process of grapes [38]. In order to evaluate if the ripening-associated accumulation of iP is a common occurrence in grapes, berries from three different grapevine cultivars, sampled from 2 weeks post flowering (wpf) to commercial harvest after 15–17 wpf, were analysed for their iP content (Fig. 1). The only other active cytokinin present in detectable amounts in grape berries, *t*Z [38], was also included in the analysis. *t*Z concentrations were generally found to be low (below 1 pmol g^−1^ fresh weight (FW)) and were elevated significantly at only one time point in Cabernet Sauvignon (Fig. 1a, 4 wpf), Riesling (Fig. 1b, 2 wpf) and Pinot Noir (Fig. 1c, 6 wpf). The biggest increase in *t*Z concentration was recorded for Pinot Noir berries (~20-fold), which, unlike Cabernet Sauvignon and Riesling berries, had not been deseeded prior to cytokinin extraction. In berries from all three cultivars tested, iP concentrations had increased significantly by four weeks after veraison (here defined as the last sampling time point prior to a significant increase in TSS levels) and continued to increase thereafter (Fig. 1). However, absolute iP concentrations at harvest varied greatly, being highest in Cabernet Sauvignon (73.9 pmol g^−1^ FW), followed by Pinot Noir (31.5 pmol g^−1^ FW) and Riesling (14.6 pmol g^−1^ FW).

**Fig. 2:**
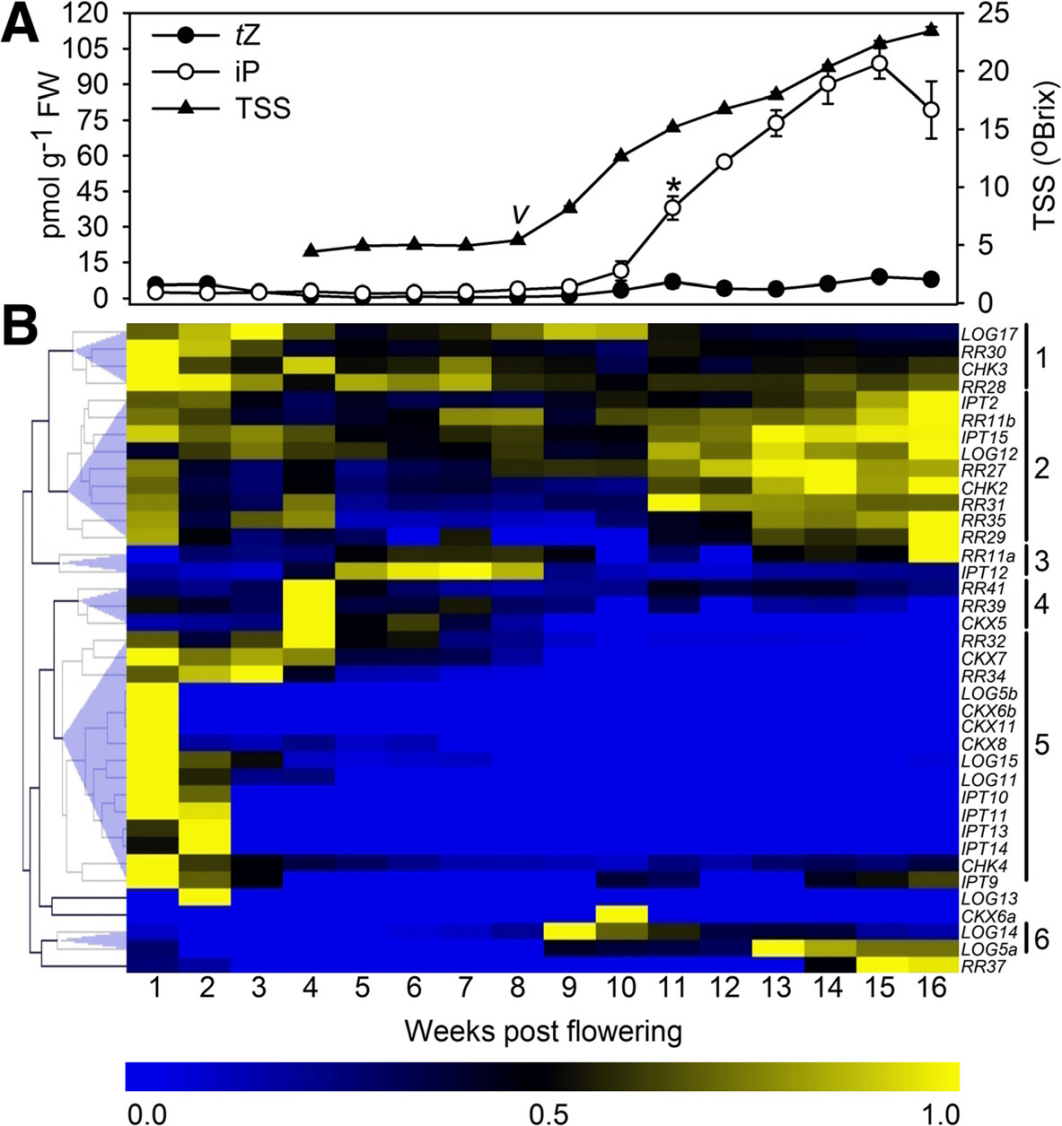
Changes in iP and *t*Z concentrations and the expression of 38 cytokinin-related genes in developing Shiraz grape berries. **a** Changes in TSS, iP and *t*Z concentrations in field-grown Shiraz berries during the 2010/2011 season. All data represent means (*n* = 3) ± SE. “*v*” indicates veraison as determined by the last time point before a significant increase (*p* <0.05) in TSS levels was recorded. The asterisk marks the start of a significant increase in iP concentrations (*p* <0.05). FW, fresh weight. **b** Heat map showing changes in transcript levels of cytokinin-related genes expressed in berries as determined by qRT-PCR. In order to adjust for differences in absolute copy numbers between the genes, the mean (*n* = 3) expression values for each transcript were normalized by dividing by the maximum copy number obtained from the berry developmental series, making all values fall between 0 and 1. Each column represents a time point after flowering, each row represents a gene of interest. Hierarchical clustering was used to group genes with similar expression profiles. Copy numbers for the 29 genes expressed at more than two time points and statistical analyses of the data are given in Additional file 5

For a more detailed analysis of cultivar-specific differences in berry iP concentrations, grapes from 13 different grapevine cultivars grown in the same vineyard were sampled at a similar TSS content (19.4–20.8°Brix) and subjected to iP quantification (Table 1). Measured iP concentrations differed up to 14-fold, ranging from 4.46 pmol g^−1^ FW in Viognier to 62.90 pmol g^−1^ FW in Shiraz, and iP abundance was not associated with berry skin colour. Whilst the iP concentration in Cabernet Sauvignon berries (Table 1) was comparable to berries in the same TSS range sampled in a different year and from a different vineyard (Fig. 1a), it was lower in berries from Riesling, Pinot Noir (Table 1 and Fig. 1b, c) and Shiraz (Table 1 and Fig. 2a).

**Table 1:** iP concentration in berries (19.4–20.8 °Brix) of 13 grape cultivars

Species	Cultivar	Colour of berry skin	iP (pmol g^−1^ FW)
*V. vinifera*	Shiraz	Red	62.90 ± 0.43^a^
*V. vinifera*	Cabernet Sauvignon	Red	40.77 ± 1.72^b^
*V. vinifera*	Durif	Red	21.85 ± 5.90^c^
*V. vinifera*	Pedro Ximénez	White	21.16 ± 1.19^cd^
Interspecific hybrid	Chambourcin	Red	20.27 ± 4.17^cd^
*V. vinifera*	Sauvignon Blanc	White	15.59 ± 4.17^cde^
*V. vinifera*	Barbera	Red	12.82 ± 0.44^def^
*V. vinifera*	Muscat Gordo Blanco	White	8.79 ± 3.83^ef^
*Vitis* hybrid	Rubired	Red	7.97 ± 0.37^ef^
*V. vinifera*	Riesling	White	6.11 ± 0.77^f^
*V. vinifera*	Pinot Noir	Red	5.69 ± 0.60^f^
*V. vinifera*	Verdelho	White	5.33 ± 0.87^f^
*V. vinifera*	Viognier	White	4.46 ± 0.77^f^

iP values represent means (*n* = 3) ± SE and different letters indicate significant differences between the cultivars as determined by one-way ANOVA (*p* <0.05) followed by Duncan’s post hoc test

### Multigene families encode grapevine genes with roles in cytokinin biosynthesis, activation, perception, signalling and catabolism

To investigate if the post-veraison increase in grape berry iP concentrations is the result of changes in local cytokinin biosynthesis, activation and/or catabolism, grapevine genes belonging to the families of isopentenyltransferases (IPTs), LONELY GUY (LOG) cytokinin nucleoside 5′-monophosphate phosphoribohydrolases and cytokinin oxidases/dehydrogenases (CKXs) were identified by sequence similarity to the respective Arabidopsis genes (Table 2, Additional files 1 and 3A-C). Cytokinin histidine kinase (CHK) receptors and type-A and –B response regulators (RRs) were also included in the analysis since a functional perception and signal transduction system is a prerequisite for the detection of, and response to, changed iP concentrations (Table 2 and Additional files 1, 3D and 4).

**Table 2:** Names, NCBI and CRIBI accession numbers and EST and splice variant numbers of the cytokinin-related grapevine sequences identified in this study

Name	NCBI Reference Sequence	NCBI ESTs	Fernandes et al. [109]^a^	CRIBI (V2) Locus ID	Splice variants	Amplified variants
VviIPT2	XM_002263711	8		VIT_206s0061g01410	1,2	1,2
VviIPT9	XM_002282976	3		VIT_219s0014g01630	1,2	1
VviIPT10	XM_002279335	0		VIT_201s0011g03640	1	1
VviIPT11	XM_002268812	0		VIT_209s0070g00710	1	1
VviIPT12	XM_002271926	2		VIT_207s0104g00270	1	1
VviIPT13	XM_003632592	0		VIT_208s0040g01010	1	1
VviIPT14	XM_002277555	4		VIT_205s0020g02630	1	1
VviIPT15	XM_002278900	5		VIT_208s0040g00100	1–5	1–5
VviLOG5a	XM_010665788	6		VIT_218s0001g00210	1	1
VviLOG5b	XM_002281803	4		VIT_203s0038g03420	1–3	1–3
VviLOG10	XM_002276739	38		VIT_218s0001g14030	1–6	1–6
VviLOG11	XM_002275378	0		VIT_208s0007g02480	1	1
VviLOG12	XM_002276243	1		VIT_208s0040g01780	1	1
VviLOG13	XM_002285210	15		VIT_206s0004g02680	1–7	1–7
VviLOG14	XM_002285680	2		VIT_206s0004g00590	1–4	1–4
VviLOG15	XM_002274711	0		VIT_213s0064g00740	1–8	1,3,5–8
VviLOG16	XM_002277816	0		VIT_208s0007g08340	1	1
VviLOG17	XM_002278269	5		VIT_204s0008g01040	1	1
VviCKX5	XM_002280761	21		VIT_218s0001g13200	1,2	1,2
VviCKX6a	XM_002270805	1		VIT_213s0158g00320	1–3	1–3
VviCKX6b	XM_002284524	1		VIT_200s0252g00040	1	1
VviCKX7	XM_002279924	15		VIT_204s0008g01880	1–3	1–3
VviCKX8	XM_002279483	0		VIT_211s0016g02110	1	1
VviCKX9	XM_003632356	0		VIT_207s0005g06025	1	1
VviCKX10	XM_002263610	1		VIT_207s0005g05960	1	1
VviCKX11	XM_002264409	0		VIT_207s0005g06010	1	1
VviCHK1	XM_002265212	2		VIT_204s0023g03680	1,2	na
VviCHK2	XM_002269941	2	VvCyt1	VIT_212s0057g00690	1–6	1–5
VviCHK3	XM_002276925	24	VvCyt2	VIT_201s0010g03780	1–4	1–4
VviCHK4	XM_002285081	10	VvCyt3	VIT_201s0011g06190	1–6	1–5
VviCHK5	XM_002271707	2		VIT_204s0069g00750	1–4	na
VviCKI	XM_002270283	0		VIT_207s0005g01380	1	na
VviRR11a	XM_002274637	2	VvRRb1	VIT_217s0000g10100	1,2	1,2
VviRR11b	XM_002267580	1	VvRRb5	VIT_201s0010g02230	1	1
VviRR25	XM_002269335	0	VvRRb2	VIT_207s0005g01010	1	1
VviRR26	XM_002270082	0	VvRRb4	VIT_211s0052g01160	1	1
VviRR27	XM_002275106	13	VvRRb6	VIT_205s0077g01480	1–4	1–4
VviRR28	XM_002281255	20		VIT_201s0011g05830	1	1
VviRR29	XM_002270797	4	VvRRb3	VIT_211s0206g00060	1,2	1,2
VviRR30	XM_002282892	8		VIT_204s0008g05900	1–4	2,4
VviRR31	FJ822980 (partial cds)	0		VIT_201s0026g00940	1	1
VviRR32	XM_002283751	9	VvRRa1	VIT_217s0000g07580	1	1
VviRR33	XM_002280710	3	VvRRa3	VIT_213s0067g03070	1	1
VviRR34	XM_002284468	1		VIT_218s0001g02540	1–4	1
VviRR35	XM_002273954	7	VvRRa2	VIT_208s0007g05390	1	1
VviRR36	XM_002266214	0		VIT_213s0067g03460	1	1
VviRR37	XM_002268316	4	VvRRa4	VIT_213s0067g03510	1	1
VviRR38	XM_002267339	0		VIT_213s0067g03450	1	1
VviRR39	XM_002267896	2		VIT_213s0067g03490	1	1
VviRR40	XM_003634849	0		VIT_213s0067g03480	1	1
VviRR41	XM_002267368	8		VIT_213s0067g03430	1	1

Phylogenetic trees for each family, using grapevine and Arabidopsis nucleotide sequences, are shown in Additional files 3 and 4. Additional file 1 contains the TAIR accession numbers of the Arabidopsis sequences used for the analyses. na, not applicable

^a^names previously used by Fernandes et al. [109]

Adenylate IPTs catalyse the initial step in the main pathway for cytokinin biosynthesis, the *N*^6^-prenylation of adenosine 5′-phosphates to form iP-riboside 5′-phosphates [51, 52]. The isoprenoid side chain can subsequently be hydroxylated by the cytochrome P450 enzymes CYP735A1/CYP735A2 to produce *t*Z-ribotides [53]. However, the single grapevine CYP735A orthologue [NCBI: XM_002280169, CRIBI: VIT_214s0006g02970] was not expressed in berries (data not shown) and cytokinin species conversion was therefore not considered to be a relevant mechanism in the context of this study. tRNA-IPTs catalyse the addition of an isopentenyl group to adenine bases in tRNAs, which can lead to the release of *c*Z and iP upon hydrolysis [54]. The grapevine genome was found to encode eight IPTs (Table 2), six of which clustered with the Arabidopsis adenylate IPTs and two orthologues (VviIPT2, VviIPT9) of the respective Arabidopsis tRNA-IPTs (Additional file 3A). Inactive cytokinin ribotides produced by the action of adenylate IPTs can be converted to active nucleobases by LOG phosphoribohydrolases [55]. Ten grapevine *LOG* genes were identified (Table 2), compared with nine genes of this family in Arabidopsis (Additional file 3B). Inactivation of cytokinins occurs by CKX-catalysed oxidative cleavage of the isoprenoid side chain [56, 57]. Out of the eight grapevine CKXs (Table 2), four were close orthologues of Arabidopsis CKXs (Additional file 3C). One-to-one orthologues were identified for all five grapevine CHK sequences (Table 2 and Additional file 3D), three of which (VviCHK2-VviCHK4) represented the *bona fide* cytokinin receptors [58]. The downstream targets of the His-Asp phosphorelay of the cytokinin signalling pathway are RRs, which are classified as negative (type-A) or positive (type-B) regulators of cytokinin signalling [59–61]. In contrast to Arabidopsis, more type-A (11) than type-B (8) RRs (Table 2 and Additional file 4) were identified in the grapevine genome.

### The expression of a subset of cytokinin-related genes coincides with the accumulation of iP during berry development

In an attempt to uncover causal relationships between the post-veraison accumulation of iP and the transcript abundance of genes involved in the control of cellular cytokinin concentrations, cytokinin nucleobases were quantified in developing Shiraz berries (Fig. 2a) and the same berry tissue was used to analyse the expression of 48 cytokinin-related genes (Table 2 and Fig. 2b). For those genes expressed at more than two time points (29), copy numbers and statistical data analyses are provided in Additional file 5. *VviCHK1*, *VviCHK5* and *VviCKI* were not included in this study due to their unclear contribution to cytokinin perception and signal transduction [62, 63]. Splice variants have been described for 40 % of the genes analysed in this study (Table 2, [64]). The primer pairs used for gene-specific amplification allowed for >90 % coverage of all known variants and were therefore expected to provide reliable expression patterns for each gene.

The changes in cytokinin concentration in Shiraz berries during development (Fig. 2a) followed a similar pattern to those observed in Cabernet Sauvignon, Riesling and Pinot Noir (Fig. 1). These results confirmed and expanded previous data obtained for a subset of the Shiraz samples using different methods of extraction and quantification [38]. *t*Z concentrations remained low and unchanged throughout development whereas a significant increase in iP concentrations was recorded from 11 wpf onwards reaching a maximum of 98.7 pmol g^−1^ FW at 15 wpf (Fig. 2a).

In total, 38 cytokinin-related genes, were found to be expressed at one or more time point(s) in berry tissue and hierarchical clustering revealed six groups of gene expression profiles (Fig. 2b). Cluster 1 contained four genes, one *LOG*, one *CHK* and two *RR*s, with the highest expression between 1 and 4 wpf and moderate to low transcript levels for the rest of development. Nine genes, composed of two *IPT*s, one *LOG*, one *CHK* and five *RR*s, constituted Cluster 2 and showed peaks of expression between 1–4 wpf and 11–16 wpf with the highest transcript abundance in the post-veraison peak. Cluster 3 was made up of *IPT12* and *RR11a*, which displayed a transcript peak between 5 and 8 wpf and, in the case of *RR11a*, also at 16 wpf. The expression in Cluster 4 (one *CKX*, two *RR*s) was mainly restricted to the 4 wpf time point. Cluster 5 was the biggest cluster, consisting of 15 genes representing all five families of cytokinin-related genes analysed, with predominant expression in very young berries (1–4 wpf). Cluster 6 contained two genes, both of them *LOG*s, which were expressed between 9 and 16 wpf. Outside of the clusters, *LOG13* and *CKX6a* transcripts were only detected at one time point (2 wpf and 10 wpf, respectively), whereas *RR37* had low expression levels in young berries (1–2 wpf) and was highly expressed from 14 to 16 wpf.

### Cytokinin-related genes are characterised by diverse expression profiles in different grapevine tissues

**Fig. 3:**
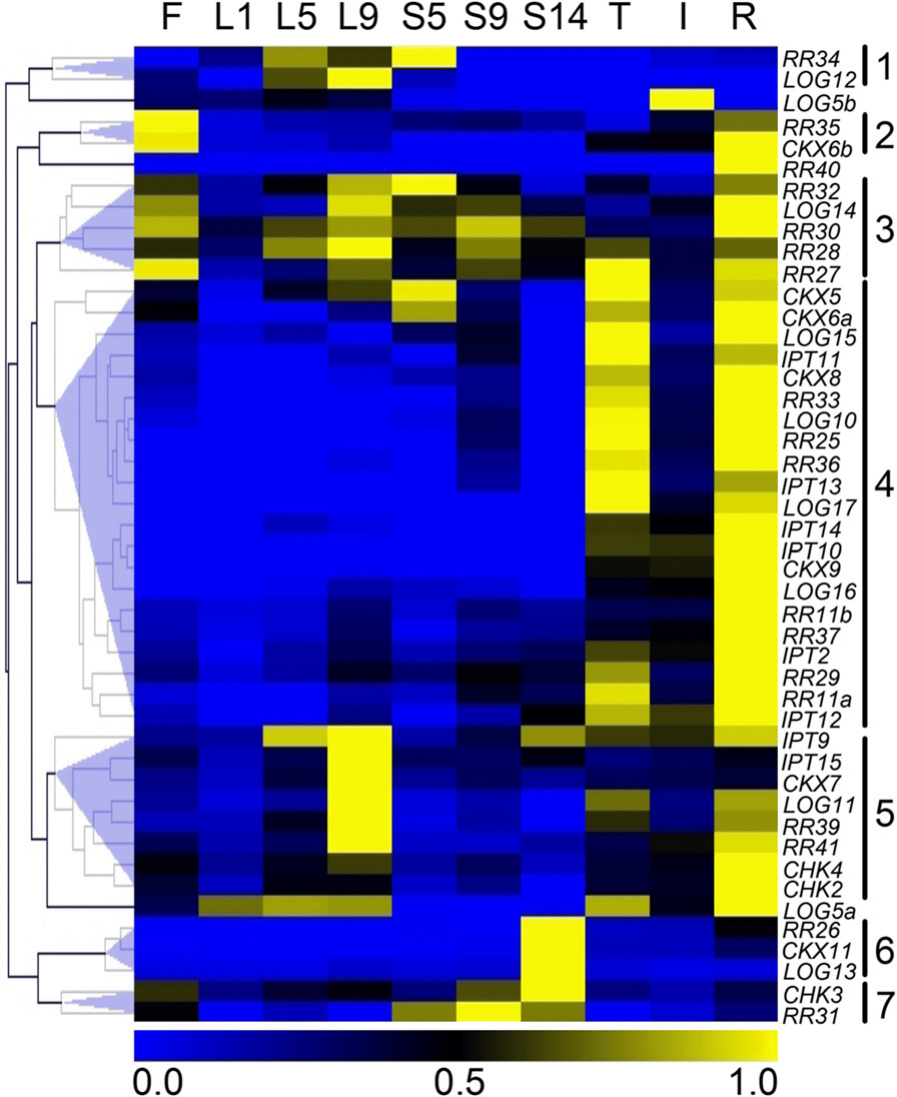
Expression profiles of 46 cytokinin-related genes in different Shiraz grapevine tissues. Heat map showing transcript levels of cytokinin-related genes expressed in different tissues of either field grown (flower, seeds, leaves, tendril, internode) or glasshouse grown (root) Shiraz plants as determined by qRT-PCR. In order to adjust for differences in absolute copy numbers between the genes, the mean (*n* = 3 technical replicates) expression values for each transcript were normalized by dividing by the maximum copy number obtained from the tissue series, making all values fall between 0 and 1. Each column represents a grapevine tissue, each row represents a gene of interest. Hierarchical clustering was used to group genes with similar expression profiles. Copy numbers for all expressed genes are given in Additional files 6 and 7. F, flower; I, internode; L, leaf (node indicated by number, increasing from the shoot apex); R, root; S, seed (wpf indicated by number); T, tendril

To gain a more complete picture of the expression and deduced activities of components of cytokinin metabolism and signalling in grapevine, the transcript accumulation of the above mentioned 48 cytokinin-related genes was also analysed in a range of other grapevine tissues (Fig. 3). All attempts to amplify *CKX10* and *RR36* fragments from any of the tested grapevine cDNAs for the generation of qRT-PCR standards failed (data not shown), so these two genes could not be included in the expression analysis. Transcripts of the remaining 46 genes, including eight genes that were not expressed in berries (Fig. 2b), were detected in at least one of the tested tissue types with gene expression profiles clustering into seven groups (Fig. 3). Cluster 1, consisting of *RR34* and *LOG12*, was characterised by predominant expression in node five (L5) and nine (L9) leaves and in seeds 5 wpf (S5; *RR34)*. Cluster 2 was also made up of two genes, *RR35* and *CKX6b*, which were expressed in flowers and roots. Cluster 3 included five genes, one *LOG* and four *RR*s, with transcripts detected in all tissues and highest expression in flowers, L9, S5, S9 or roots. The largest set of genes (21) was grouped in Cluster 4 and was predominantly expressed in tendrils and roots. *CKX5* and *CXK6a* were also highly expressed in S5. Cluster 5 contained eight genes, representing all five families of cytokinin-related genes analysed, with highest expression in L9 or roots. The common feature of *RR26*, *CKX11* and *LOG13* in Cluster 6 was S14-specific expression, whereas Cluster 7 *CHK3* and *RR31* transcripts were mainly detected in flowers and seeds. Three genes showed unique expression profiles: *LOG5b* was mainly expressed in internodes, *LOG5a* showed expression in all tissues except seeds and *RR40* transcripts were only detected in roots. Copy numbers of all expressed genes are provided in Additional files 6 and 7.

### A ripening-associated increase in iP concentrations also occurs in tomato and strawberry

**Fig. 4:**
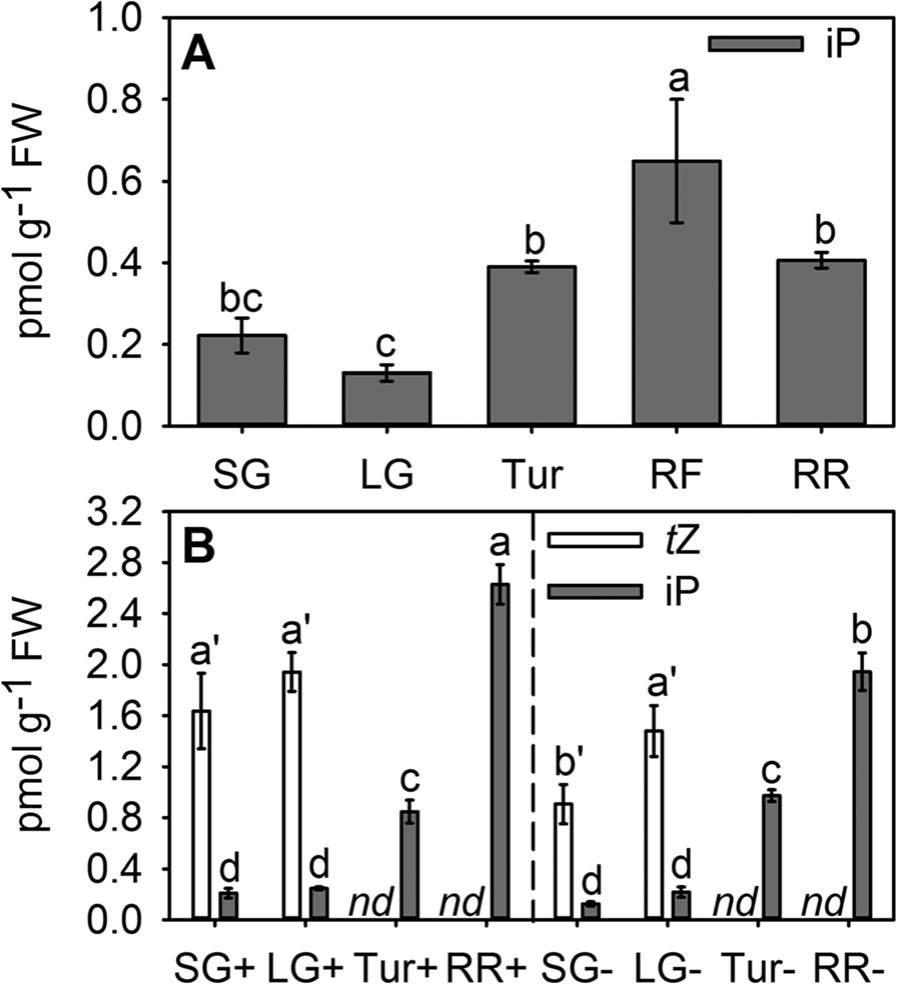
Concentrations of iP and *t*Z in developing tomatoes and strawberries. iP and *t*Z were analysed by LC-MS/MS in (**a**) small green (SG), large green (LG), turning (Tur), red firm (RF) and red ripe (RR) tomatoes and in (**b**) small green (SG), large green (LG), turning (Tur) and red ripe (RR) strawberry receptacles with (+) and without (−) achenes. *t*Z concentrations were below the limit of quantification in tomato. FW, fresh weight; *nd*, not detected. Bars represent means ± SE (*n* = 3) and are denoted by a different letter (a-d, iP; a’-b’, *t*Z) if the means for each time point differed significantly (*p* <0.05) using one-way ANOVA followed by Duncan’s post hoc test

Studies involving the measurement of cytokinins throughout fruit development are scarce, which could be one reason why the accumulation of iP during the ripening phase of fruit has not been reported from any fruit species other than grape [38]. In order to investigate if the ripening-associated iP increase is unique to grape berries or a common phenomenon in fruit, nucleobase cytokinins were measured in several developmental stages of tomato and strawberry fruit (Fig. 4). In tomato, *t*Z concentrations were generally below the limit of quantification and iP concentrations were below 1 pmol g^−1^ FW in all stages tested (Fig. 4a). However, in red firm fruit, the iP concentration was found to be significantly increased. In strawberry, *t*Z could only be detected in receptacles of pre-ripening fruit (Fig. 4b). In small green fruit the concentration of *t*Z was significantly decreased by the removal of achenes prior to cytokinin extraction. Similar to tomato, iP concentrations in strawberry receptacles were low, but were found to be significantly increased in turning fruit and were even higher in fully mature, red ripe strawberries (Fig. 4b). At this last developmental stage, achene-containing receptacles contained significantly higher concentrations of iP than receptacles without achenes.

## Discussion

Most of the published studies on cytokinins in fruit, including grape [23–25], strawberry [20], tomato [19], apple [65], watermelon (*Citrullus lanatus* (Thunb.) Mansf.) [66], Japanese pear (*Pyrus serotina* L.) [67] and persimmon [68], have utilized bioassays, based on changes in cell proliferation or pigment accumulation, to determine the concentration of active cytokinins. Across all fruit species, high cytokinin activity was reported in young fruit progressing through the cell division phase, whereas activities were low or undetectable in ripening fruit. This seems to contradict the ripening-associated increase in iP concentrations reported for four grapevine cultivars (Figs. 1 and 2a), tomato and strawberry (Fig. 4) in this work, but it has to be considered that the above mentioned bioassays were mostly using *t*Z, and never iP, as the reference cytokinin. Detectable *t*Z concentrations were found to be restricted to pre-ripening strawberries (Fig. 4) and in pre-veraison grapes, seeds seemed to be the main *t*Z source as evidenced by a high *t*Z concentration in seed-containing Pinot Noir berry tissue at 6 wpf (Fig. 1c). The accumulation of *t*Z during early grape seed development has previously been reported [69, 70]. Although both, *t*Z and iP, are classified as cytokinins and only differ in the hydroxylation of the side chain, they need to be considered as different and independent molecules in regard to their localization and transport within the plant, signalling outputs and biological effects. In Arabidopsis, recent experiments with mutants impaired in the *trans*-hyroxylation step that converts iP to *t*Z have revealed that the regulation of cell proliferation in the shoot apical meristem is a function exclusive to *t*Z [71]. In further support of a functional specification, Takei et al. [9] have reported that application of Z-type cytokinins to maize (*Zea mays* L.) leaves led to the induction of *ZmRR1*, whereas no changes in *ZmRR1* expression were observed in response to iP-type cytokinins. In addition, CHK receptors [72–75] and members of the CKX degradation pathway [57, 76] were reported to differ in their preference for iP and *t*Z. A different role for *t*Z and iP in the long distance signalling pathways of plants has long been discussed since xylem sap has been found to mainly contain *t*Z in the form of its ribosides and ribotides [9, 77, 78], whereas iP ribosides and ribotides seem to be transported through the phloem [78, 79]. From the evidence listed above it is therefore feasible that changes in fruit iP concentrations have previously escaped detection due to lack of activity of this cytokinin in the chosen bioassays. However, from the few examples where iP has been quantified throughout the development of fleshy fruit, grapes ([38]; this study) were shown to accumulate up to 100-fold more iP during the ripening phase than tomato (this study), strawberry (this study) and kiwifruit [21, 37] and no increase in iP concentration was detected during the transition from pink to red raspberries [22]. iP concentrations in tomato, strawberry and kiwifruit fall into a similar range to what has been published for Arabidopsis seedlings [80, 81], maize roots, leaves and kernels [82], young ‘Microtom’ tomato ovaries [83], rice inflorescence meristem [14] and various soybean (*Glycine max* (L.) Merr.) tissues [84], whereas the iP quantities detected in grape berries are unprecedented. This points to a specific relevance for iP accumulation in grapes and might be related to the expansion-driven post-veraison growth and the high rate of sugar accumulation in these berries [85]. A study utilizing data from eight independent Arabidopsis microarray experiments revealed the induction of 12 expansins and 18 other cell-wall-related genes by cytokinins [86], confirming previously reported cytokinin-induced changes of cell wall characteristics, such as increased extensibility [87], or decreased thickness [88]. It is therefore possible that the post-veraison expansion of berry cells is at least in part controlled by the observed changes in iP concentrations. The induction of cell wall invertase genes and the large number of cytokinin-regulated genes involved in trehalose-6-phosphate metabolism [86] further indicate a possible role for iP in the maintenance of sink strength in ripening berries. Cytokinins are known as positive regulators of sink strength in vegetative organs, attracting carbohydrates and amino acids from source tissues to sites of high cytokinin concentration [89–92]. Studies on *Chenopodium rubrum* L. cell suspension cultures [93] and leaf senescence in tobacco (*Nicotiana tabacum* L.) [4, 94] have suggested that sink strength is likely to be mediated by cytokinin-inducible cell wall invertases and hexose transporters, which are functionally linked to the apoplastic phloem unloading pathway and hence to the maintenance of a sucrose gradient between source and sink organs [95]. In grapes, a shift from symplastic to apoplastic phloem unloading, coinciding with the start of the ripening phase and the increased expression of invertases and hexose transporters, has been described [96, 97]. In support of a possible role of iP in the maintenance of post-veraison berries as strong sink organs, a cell wall invertase gene with an expression profile resembling the post-veraison pattern of iP accumulation has been reported in Cabernet Sauvignon [98, 99].

The causal connection for the large variation in maximal iP concentration between different grapevine cultivars observed in this study (Figs. 1 and 2a, Table 1) is unknown and will require further investigation, but genetic as well as environmental factors are likely contributors. The well-described stimulatory effect of cytokinins on anthocyanin accumulation in a number of plant species [100–102] suggested a possible link between the post-veraison accumulation of iP and anthocyanins in red cultivars. However, iP data obtained from red and white skinned cultivars at a similar berry sugar level, showed that, although the three cultivars with the highest iP concentrations were red skinned, a clear distinction between red and white skinned cultivars could not be made. For example, the iP concentration of Rubired berries, which in addition to the skin also produce anthocyanins in the flesh, could not be distinguished from white cultivars with low iP concentrations, e.g. Riesling or Viognier (Table 1).

A number of cytokinin nucleobases, ribosides and ribotides, including low levels of iP-type cytokinins, have been detected in the bleeding sap of Shiraz vines at budbreak [103] and it cannot be excluded that the post-veraison iP accumulation reported in this study (Figs. 1 and 2a) was the result of iP import from the phloem. However, the spatial expression patterns of cytokinin-related genes in tomato [83] and kiwifruit [37] indicated that local cytokinin biosynthesis and degradation occur in fruit and play an important role in fruit development. This was also confirmed in grapes, where genes regulating cytokinin biosynthesis (*IPT*s), activation (*LOG*s), degradation (*CKX*s), perception (*CHK*s) and signalling (*RR*s) were found to be expressed in all stages of berry development (Fig. 2b, Additional file 5). Transcripts of all eight grapevine *IPT*s (Table 2) were detected in berries. Five of them (*IPT10*-*14*) were restricted to pre-veraison stages, the other three (*IPT2*, *IPT9*, *IPT15*) were expressed pre- and post-veraison, including during the time of iP accumulation (Fig. 2). The expression of specific *IPT* genes at certain developmental stages seems to be highly regulated since *IPT12*, which peaked between 5 and 8 wpf, has been described as the target of two siRNAs in post-veraison berries leading to post-transcriptional silencing [104]. The increased expression of the two tRNA-IPTs (*IPT2*, *IPT9*) in post-veraison berries might reflect a bigger contribution of tRNA-hydrolysis to the cytokinin pool in these later stages of berry development, which could produce *c*Z and iP [54]. However, as was the case in a previous study [38], *c*Z concentrations in Shiraz berries remained below the detection limit throughout berry development (data not shown). Judging from the expression of *IPT* genes in other grapevine organs (Fig. 3 and Additional file 6) and in agreement with reports from Arabidopsis [105], tomato [83] and soybean [84], local cytokinin biosynthesis seemed to occur throughout the plant, in particular in roots, tendrils, and mature leaves. The LOG-dependent pathway of producing active cytokinin nucleobases from ribotide precursors has recently been established as the dominant cytokinin-activating mechanism in rice [55] and Arabidopsis [106]. It also appeared to be active early (1–3 wpf) and late (9–16 wpf) in berry development, since *LOG12* and *LOG17* were expressed in pre- and post-veraison fruit, four additional *LOG*s were expressed during the pre-veraison stages and expression of *LOG5a* and *LOG14* was post-veraison-specific (Fig. 2b) with the transcript accumulation of *LOG5a* closely matching the pattern of iP increase (Fig. 2). All ten *LOG* genes (Table 2) were found to be expressed with distinct patterns in at least one of the grapevine tissues tested, with predominant transcript accumulation in the same organs as *IPT*s (Fig. 3 and Additional file 6).

The irreversible degradation of cytokinins by CKX enzymes is a vital part of the regulation of local cytokinin concentrations [107] and in grape berries seemed to be restricted to early developmental stages (1–4 wpf, Fig. 2a). The progressive decrease of *CKX5* transcripts has previously been reported in two microarray studies investigating transcriptional changes in developing grape berries [99, 108]. The lack of cytokinin degradation in post-veraison grapes might contribute to the large increase in iP concentrations, especially since iP has been found to be more susceptible to CKX-catalysed degradation than other cytokinins [57, 76].

All three grapevine cytokinin receptor genes (Table 2) were expressed in every tissue (Fig. 3 and Additional file 6) and berry developmental stage analysed (Fig. 2b), but whilst *CHK3* and *CHK4* showed higher transcript accumulation in pre-veraison berries, *CHK2* was characterised by a significant increase in expression during the late, high-iP, post-veraison phase. The Arabidopsis orthologue of VviCHK2 has been reported to preferentially bind iP, whereas the other two receptors preferred *t*Z [74]. The post-veraison increase in expression of *CHK2* might therefore represent an amplifier for the orchestration of iP-specific responses during the ripening phase. Supporting this hypothesis is the expression of a set of post-veraison-specific *RR*s, including four B-type *RR*s (*RR11a*, *RR11b*, *RR27*, *RR29*) and three A-type *RR*s (*RR31*, *RR35*, *RR37*) which could translate the iP signal into a ripening-specific, transcriptional response (Table 2 and Fig. 2b). Pre-veraison berries were characterised by the expression of a separate set of *RR* genes (two B-type *RR*s, four A-type *RR*s), whereas no *RR* gene with significant transcript accumulation in both pre- and post-veraison berry stages was identified (Table 2 and Fig. 2b). In other grapevine organs, roots showed the overall highest expression of *RR*s, but *RR* transcripts were found in all tested tissues, with nine *RR*s expressed ubiquitously and nine *RR*s restricted to specific organs (Fig. 3 and Additional file 7).

## Conclusions

The present study provides evidence for the occurrence of a ripening-associated increase in iP concentrations in a number of different grapevine cultivars, strawberry and tomato and therefore suggests a universal role for this cytokinin in the regulation of fruit ripening processes. The unusually high concentrations of iP found in post-veraison grape berries suggest a specific relevance for iP accumulation in these fruit, possibly related to the equally high concentrations of sugar stored in grapes. Developmental changes in the expression of genes related to cytokinin biosynthesis, activation, perception, signalling and catabolism indicate that the regulation of berry cytokinin concentrations and the response to specific cytokinin species can be controlled locally and provide a possible explanation for the post-veraison accumulation of iP. Distinct expression patterns within each gene family in berries and a range of other grapevine tissues suggest spatial and temporal specification and hence a highly complex system for the regulation of cytokinin concentrations and responses.

## Availability of supporting data

All supporting data are included as additional files.

## Additional files

Additional file 1:
**TAIR accession numbers of the Arabidopsis nucleotide sequences used for phylogenetic analyses.** (PDF 33 kb) Additional file 2:
**Gene-specific primer pairs used for qRT-PCR analyses.** (PDF 59 kb) Additional file 3:
**Phylogenetic relationship of** ***IPT*** **,** ***LOG*** **,** ***CKX*** **and** ***CHK*** **coding sequences from grapevine and Arabidopsis.** Unrooted trees of (A) *IPT*, (B) *LOG*, (C) *CKX* and (D) *CHK* sequences were generated from alignments created with MUSCLE [45], all positions containing gaps and missing data were eliminated. The evolutionary history was inferred by using the Maximum Likelihood method based on the JTT matrix-based model [46]. A bootstrap consensus tree was generated from 100 replicates [47] and branches corresponding to partitions replicated in less than 70 % replicates were collapsed. Initial tree(s) for the heuristic search were obtained automatically by applying Neighbor-Join and BioNJ algorithms to a matrix of pairwise distances estimated using a JTT model and then selecting the topology with superior log value. The coding data was translated assuming a standard genetic code table. The naming of grapevine genes followed the guidelines published by Grimplet et al. [48]. Grapevine sequences are highlighted with a grey background. NCBI or TAIR accession numbers for all sequences used in the phylogenetic analysis are listed in Table 2 and Additional file 1. (PDF 52 kb) Additional file 4:
**Phylogenetic relationship of** ***RR*** **coding sequences from grapevine and Arabidopsis.** The unrooted tree was generated from an alignment created with MUSCLE [45], all positions containing gaps and missing data were eliminated. The evolutionary history was inferred by using the Maximum Likelihood method based on the JTT matrix-based model [46]. A bootstrap consensus tree was generated from 100 replicates [47] and branches corresponding to partitions replicated in less than 70 % replicates were collapsed. Initial tree(s) for the heuristic search were obtained automatically by applying Neighbor-Join and BioNJ algorithms to a matrix of pairwise distances estimated using a JTT model and then selecting the topology with superior log value. The coding data was translated assuming a standard genetic code table. The naming of grapevine genes followed the guidelines published by Grimplet et al. [48]. Grapevine sequences are highlighted with a grey background. NCBI or TAIR accession numbers for all sequences used in the phylogenetic analysis are listed in Table 2 and Additional file 1. (PDF 31 kb) Additional file 5:
**Transcript accumulation of cytokinin-related genes expressed at two or more time points in a Shiraz berry developmental series.** The expression of (A) *IPT*, (B) *LOG*, (C) *CKX*, (D) *RR* and (E) pre-veraison-specific genes was analysed by qRT-PCR. All data represent means (*n* = 3) ± SE and LSD values were determined at the *p* <0.05 significance level. Asterisks mark samples in which expression could not be detected. (PDF 188 kb) Additional file 6:
**Transcript accumulation of** ***IPT*** **,** ***LOG*** **,** ***CKX*** **and** ***CHK*** **genes in different Shiraz tissues.** The expression of (A) *IPT*, (B) *LOG*, (C) *CKX* and (D) *CHK* genes was analysed by qRT-PCR. All data represent means ± SE (*n* = 3 technical replicates). Asterisks mark tissues in which expression could not be detected. F, flower; I, internode; L, leaf (node indicated by number); R, root; S, seed (wpf indicated by number); T, tendril. (PDF 493 kb) Additional file 7:
**Transcript accumulation of** ***RR*** **genes in different Shiraz tissues.** The expression of *RR* genes was analysed by qRT-PCR. All data represent means ± SE (*n* = 3 technical replicates). Asterisks mark tissues in which expression could not be detected. F, flower; I, internode; L, leaf (node indicated by number); R, root; S, seed (wpf indicated by number); T, tendril. (PDF 310 kb)

## Abbreviations

ACT: Actin
ANOVA: Analysis of variance
CHK: Cytokinin histidine kinase
CKX: Cytokinin oxidase/dehydrogenase
CPPU: N-(2-Chloro-4-pyridinyl)-N’-phenylurea
ESI: Electrospray ionization
F: Flower
FW: Fresh weight
HPLC: High performance liquid chromatography
I: Internode
iP: *N*^*6*^-(Δ^2^-Isopentenyl)-adenine
IPT: Isopentenyltransferase
L: Leaf
LC-MS: Liquid chromatography-mass spectrometry
LG: Large green (tomato ripening stage)
LOG: LONLEY GUY
LSD: Least significant difference
MS/MS: Tandem mass spectrometry
NA: Not applicable
ND: Not detected
qRT-PCR: Quantitative real time polymerase chain reaction
R: Root
RF: Red firm (tomato ripening stage)
RR: Response regulator or Red ripe (tomato ripening stage)
S: Seed
SE: Standard error
SG: Small green (tomato ripening stage)
SPE: Solid phase extraction
T: Tendril
TDZ: Thidiazuron
TSS: Total soluble solids
Tur: Turning (tomato ripening stage)
WPF: Weeks post flowering
*c*Z: *cis*-Zeatin
*t*Z: *trans*-Zeatin

## References

[1] Mok DW, Mok MC (2001). Cytokinin metabolism and action. Annu Rev Plant Physiol Plant Mol Biol.

[2] Sakakibara H (2006). Cytokinins: activity, biosynthesis, and translocation. Annu Rev Plant Biol.

[3] Amasino R (2005). 1955: Kinetin arrives. The 50^th^ anniversary of a new plant hormone. Plant Physiol.

[4] Gan S, Amasino RM (1995). Inhibition of leaf senescence by autoregulated production of cytokinin. Science.

[5] Kim HJ, Ryu H, Hong SH, Woo HR, Lim PO, Lee IC (2006). Cytokinin-mediated control of leaf longevity by AHK3 through phosphorylation of ARR2 in *Arabidopsis*. Proc Natl Acad Sci U S A.

[6] Werner T, Motyka V, Laucou V, Smets R, Van Onckelen H, Schmülling T (2003). Cytokinin-deficient transgenic Arabidopsis plants show multiple developmental alterations indicating opposite functions of cytokinins in the regulation of shoot and root meristem activity. Plant Cell.

[7] Werner T, Motyka V, Strnad M, Schmülling T (2001). Regulation of plant growth by cytokinin. Proc Natl Acad Sci.

[8] Samuelson ME, Larsson C-M (1993). Nitrate regulation of zeation riboside levels in barley roots: Effects of inhibitors of N assimilation and comparison with ammonium. Plant Sci.

[9] Takei K, Sakakibara H, Taniguchi M, Sugiyama T (2001). Nitrogen-dependent accumulation of cytokinins in root and the translocation to leaf: Implication of cytokinin species that induces gene expression of maize response regulator. Plant Cell Physiol.

[10] Argueso CT, Ferreira FJ, Kieber JJ (2009). Environmental perception avenues: the interaction of cytokinin and environmental response pathways. Plant Cell Environ.

[11] Cooper JB, Long SR (1994). Morphogenetic rescue of *Rhizobium meliloti* nodulation mutants by trans-zeatin secretion. Plant Cell.

[12] Sasaki T, Suzaki T, Soyano T, Kojima M, Sakakibara H, Kawaguchi M. Shoot-derived cytokinins systemically regulate root nodulation. Nat Commun. 2014;5:4983 doi:10.1038/ncomms5983. 10.1038/ncomms5983 25236855

[13] Kudo T, Kiba T, Sakakibara H (2010). Metabolism and long-distance translocation of cytokinins. J Integr Plant Biol.

[14] Ashikari M, Sakakibara H, Lin S, Yamamoto T, Takashi T, Nishimura A (2005). Cytokinin oxidase regulates rice grain production. Science.

[15] Bartrina I, Otto E, Strnad M, Werner T, Schmülling T (2011). Cytokinin regulates the activity of reproductive meristems, flower organ size, ovule formation, and thus seed yield in *Arabidopsis thaliana*. Plant Cell.

[16] Quesnelle PE, Emery RJN (2007). cis-Cytokinins that predominate in *Pisum sativum* during early embryogenesis will accelerate embryo growth *in vitro*. Can J Bot.

[17] Emery RJN, Ma Q, Atkins CA (2000). The forms and sources of cytokinins in developing white lupine seeds and fruits. Plant Physiol.

[18] Tarkowski P, Tarkowská D, Novák O, Mihaljević S, Magnus V, Strnad M (2006). Cytokinins in the perianth, carpels, and developing fruit of *Helleborus niger* L. J Exp Bot.

[19] Desai N, Chism GW (1978). Changes in cytokinin activity in the ripening tomato fruit. J Food Sci.

[20] Lis EK, Borkowska B, Antoszewski R (1978). Growth regulators in the strawberry fruit. Fruit Science Reports.

[21] Lewis DH, Burge GK, Hopping ME, Jameson PE (1996). Cytokinins and fruit development in the kiwifruit (*Actinidia deliciosa*). II. Effects of reduced pollination and CPPU application. Physiol Plant.

[22] Miret J, Cela J, Bezerra L, Arrom L, Juvany M, Müller M (2014). Application of a rapid and sensitive method for hormonal and vitamin E profiling reveals crucial regulatory mechanisms in flower senescence and fruit ripening. J Plant Growth Regul.

[23] Alleweldt G, Duering H, Waits G (1975). Untersuchungen zum mechanismus der zuckereinlagerung in die wachsenden weinbeeren. Angew Bot.

[24] Chacko EK, Saidha T, Swamy RD, Reddy YN, Kohli RR (1976). Studies on cytokinins in fruits 1: occurrence and levels of cytokinin-like substances in grape berries at different developmental stages. Vitis.

[25] Inaba A, Ishida M, Sobajima Y (1976). Changes in endogenous hormone concentrations during berry development in relation to the ripening of Delaware grapes. J Jpn Soc Hortic Sci.

[26] Gillaspy G, Ben-David H, Gruissem W (1993). Fruits - a developmental perspective. Plant Cell.

[27] Srivastava A, Handa A (2005). Hormonal regulation of tomato fruit development: a molecular perspective. J Plant Growth Regul.

[28] Zabadal TJ, Bukovac MJ (2006). Effect of CPPU on fruit development of selected seedless and seeded grape cultivars. HortSci.

[29] Kim JG, Takami Y, Mizugami T, Beppu K, Fukuda T, Kataoka I (2006). CPPU application on size and quality of hardy kiwifruit. Sci Hortic.

[30] NeSmith DS (2002). Response of rabbiteye blueberry (*Vaccinium ashei* Reade) to the growth regulators CPPU and gibberellic acid. HortSci.

[31] Stern RA, Ben-Arie R, Neria O, Flaishman M (2003). CPPU and BA increase fruit size of ‘Royal Gala’ (*Malus domestica*) apple in a warm climate. Journal of Horticultural Science and Biotechnology.

[32] Flaishman MA, Shargal A, Stern RA (2001). The synthetic cytokinin CPPU increases fruit size and yield of ‘Spadona’ and ‘Costia’ pear (*Pyrus communis* L.). J Hortic Sci Biotechnol.

[33] Davies C, Böttcher C, Nath P, Bouzayen M, Matto A, Pech JC (2014). Other hormone signals during ripening. Fruit ripening: physiology, signalling and genomics.

[34] Peppi MC, Fidelibus MW (2008). Effects of forchlorfenuron and abscisic acid on the quality of ‘Flame Seedless’ grapes. HortSci.

[35] Famiani F, Battistelli A, Moscatello S, Boco M, Antognozzi E (1999). Thidiazuron affects fruit growth, ripening and quality of *Actinidia deliciosa*. J Hortic Sci Biotechnol.

[36] Itai A, Tanabe K, Tamura F, Susaki S, Yonemori K, Sugiura A (1995). Synthetic cytokinins control persimmon fruit shape, size and quality. J Hortic Sci.

[37] Pilkington SM, Montefiori M, Galer AL, Emery RJN, Allan AC, Jameson PE (2013). Endogenous cytokinin in developing kiwifruit is implicated in maintaining fruit flesh chlorophyll levels. Ann Bot.

[38] Böttcher C, Boss PK, Davies C (2013). Increase in cytokinin levels during ripening in developing *Vitis vinifera* cv. Shiraz berries. Am J Enol Vitic.

[39] Böttcher C, Burbidge CA, di Rienzo V, Boss PK, Davies C (2015). Jasmonic acid-isoleucine formation in grapevine (*Vitis vinifera* L.) by two enzymes with distinct transcription profiles. J Integr Plant Biol.

[40] Kalua CM, Boss PK (2009). Evolution of volatile compounds during the development of Cabernet Sauvignon grapes (*Vitis vinifera* L.). J Agric Food Chem.

[41] Kalua CM, Boss PK (2010). Comparison of major volatile compounds from Riesling and Cabernet Sauvignon grapes (*Vitis vinifera* L.) from fruitset to harvest. Aust J Grape Wine Res.

[42] Böttcher C, Keyzers RA, Boss PK, Davies C (2010). Sequestration of auxin by the indole-3-acetic acid-amido synthetase GH3-1 in grape berry (*Vitis vinifera* L.) and the proposed role of auxin conjugation during ripening. J Exp Bot.

[43] Davies C, Nicholson EL, Böttcher C, Burbidge CA, Bastian SEP, Harvey KE (2015). Shiraz wines made from grape berries (*Vitis vinifera*) delayed in ripening by plant growth regulator treatment have elevated rotundone concentrations and “pepper” flavor and aroma. J Agric Food Chem.

[44] Tamura K, Stecher G, Peterson D, Filipski A, Kumar S (2013). MEGA6: molecular evolutionary genetics analysis version 6.0. Mol Biol Evol.

[45] Edgar RC (2004). MUSCLE: multiple sequence alignment with high accuracy and high throughput. Nucleic Acids Res.

[46] Jones DT, Taylor WR, Thornton JM (1992). The rapid generation of mutation data matrices from protein sequences. Comput Appl Biosci.

[47] Felsenstein J (1985). Confidence limits on phylogenies: an approach using the bootstrap. Evolution.

[48] Grimplet J, Adam-Blondon A-F, Bert P-F, Bitz O, Cantu D, Davies C (2014). The grapevine gene nomenclature system. BMC Genomics.

[49] Böttcher C, Burbidge CA, Boss PK, Davies C (2013). Interactions between ethylene and auxin are crucial to the control of grape (*Vitis vinifera* L.) berry ripening. BMC Plant Biol.

[50] Böttcher C, Boss PK, Davies C (2011). Acyl substrate preferences of an IAA-amido synthetase account for variations in grape (*Vitis vinifera* L.) berry ripening caused by different auxinic compounds indicating the importance of auxin conjugation in plant development. J Exp Bot.

[51] Kakimoto T (2001). Identification of plant cytokinin biosynthetic enzymes as dimethylallyl diphosphate:ATP/ADP Isopentenyltransferases. Plant Cell Physiol.

[52] Takei K, Sakakibara H, Sugiyama T (2001). Identification of genes encoding adenylate isopentenyltransferase, a cytokinin biosynthesis enzyme, in *Arabidopsis thaliana*. J Biol Chem.

[53] Takei K, Yamaya T, Sakakibara H (2004). *Arabidopsis CYP735A1* and *CYP735A2* encode cytokinin hydroxylases that catalyze the biosynthesis of *trans*-zeatin. J Biol Chem.

[54] Murai N, Mok DWS, Mok MC (1994). Cytokinin biosynthesis in tRNA and cytokinin incorporation into plant RNA. Cytokinins: chemistry, activity, and function.

[55] Kurakawa T, Ueda N, Maekawa M, Kobayashi K, Kojima M, Nagato Y (2007). Direct control of shoot meristem activity by a cytokinin-activating enzyme. Nature.

[56] Hare PD, van Staden J (1994). Cytokinin oxidase: biochemical features and physiological significance. Physiol Plant.

[57] Galuszka P, Popelková H, Werner T, Frébortová J, Pospíšilová H, Mik V (2007). Biochemical characterization of cytokinin oxidases/dehydrogenases from *Arabidopsis thaliana* expressed in *Nicotiana tabacum* L. J Plant Growth Regul.

[58] Hwang I, Sheen J (2001). Two-component circuitry in *Arabidopsis* cytokinin signal transduction. Nature.

[59] D’Agostino IB, Kieber JJ (1999). Phosphorelay signal transduction: the emerging family of plant response regulators. Trends Biochem Sci.

[60] D’Agostino IB, Deruère J, Kieber JJ (2000). Characterization of the response of the Arabidopsis response regulator gene family to cytokinin. Plant Physiol.

[61] Imamura A, Hanaki N, Nakamura A, Suzuki T, Taniguchi M, Kiba T (1999). Compilation and characterization of *Arabiopsis thaliana* response regulators implicated in His-Asp phosphorelay signal transduction. Plant Cell Physiol.

[62] Yamada H, Suzuki T, Terada K, Takei K, Ishikawa K, Miwa K (2001). The Arabidopsis AHK4 histidine kinase is a cytokinin-binding receptor that transduces cytokinin signals across the membrane. Plant Cell Physiol.

[63] Choi J, Hwang I (2007). Cytokinin: perception, signal transduction, and role in plant growth and development. J Plant Biol.

[64] Vitulo N, Forcato C, Carpinelli EC, Telatin A, Campagna D, D’Angelo M (2014). A deep survey of alternative splicing in grape reveals changes in the splicing machinery related to tissue, stress condition and genotype. BMC Plant Biol.

[65] Letham DS, Williams MW (1969). Regulators of cell division in plant tissues VIII. The cytokinins of the Apple fruit. Physiol Plant.

[66] Prakash R, Maheshwari SC (1970). Studies on cytokinins in Watermelon seeds. Physiol Plant.

[67] Ohkawa M (1973). Studies on growth of Japanese pear fruits. I. Isolation of zeatin and its related compound from immature Japanese pear fruits. J Jpn Soc Hortic Sci.

[68] Sobajima Y, Ishida M, Inaba A, Horiguchi H (1974). Studies on the fruit development of Japanese persimmon (*Diospyros kaki* L.) I. Cytokinin activity in young fruits. J Jpn Soc Hortic Sci.

[69] Pandey SN, Singh R (1989). Endogenous level of hormones in developing grape seed *Vitis vinifera* Linn. Indian J Plant Physiol.

[70] Zhang X, Luo G, Wang R, Wang J, Himelrick DG (2003). Growth and developmental responses of seeded and seedless grape berries to shoot girdling. J Am Soc Hortic Sci.

[71] Kiba T, Takei K, Kojima M, Sakakibara H (2013). Side-chain modification of cytokinins controls shoot growth in *Arabidopsis*. Dev Cell.

[72] Romanov GA, Lomin SN, Schmülling T (2006). Biochemical characteristics and ligand-binding properties of *Arabidopsis* cytokinin receptor AHK3 compared to CRE1/AHK4 as revealed by a direct binding assay. J Exp Bot.

[73] Stolz A, Riefler M, Lomin SN, Achazi K, Romanov GA, Schmülling T (2011). The specificity of cytokinin signalling in *Arabidopsis thaliana* is mediated by differing ligand affinities and expression profiles of the receptors. Plant J.

[74] Shi X, Rashotte A (2012). Advances in upstream players of cytokinin phosphorelay: receptors and histidine phosphotransfer proteins. Plant Cell Rep.

[75] Choi J, Lee J, Kim K, Cho M, Ryu H, An G (2012). Functional identification of *OsHk6* as a homotypic cytokinin receptor in rice with preferential affinity for iP. Plant Cell Physiol.

[76] Bilyeu KD, Cole JL, Laskey JG, Riekhof WR, Esparza TJ, Kramer MD (2001). Molecular and biochemical characterization of a cytokinin oxidase from Maize. Plant Physiol.

[77] Beveridge CA, Murfet IC, Kerhoas L, Sotta B, Miginiac E, Rameau C (1997). The shoot controls zeatin riboside export from pea roots. Evidence from the branching mutant *rms4*. Plant J.

[78] Hirose N, Takei K, Kuroha T, Kamada-Nobusada T, Hayashi H, Sakakibara H (2008). Regulation of cytokinin biosynthesis, compartmentalization and translocation. J Exp Bot.

[79] Corbesier L, Prinsen E, Jacqmard A, Lejeune P, Van Onckelen H, Périlleux C (2003). Cytokinin levels in leaves, leaf exudate and shoot apical meristem of *Arabidopsis thaliana* during floral transition. J Exp Bot.

[80] Kasahara H, Takei K, Ueda N, Hishiyama S, Yamaya T, Kamiya Y (2004). Distinct isoprenoid origins of *cis*- and *trans*-zeatin biosyntheses in *Arabidopsis*. J Biol Chem.

[81] Zhang X, Chen Y, Lin X, Hong X, Zhu Y, Li W (2013). Adenine phosphoribosyl transferase 1 is a key enzyme catalyzing cytokinin conversion from nucleobases to nucleotides in *Arabidopsis*. Mol Plant.

[82] Veach YK, Martin RC, Mok DWS, Malbeck J, Vankova R, Mok MC (2003). *O*-Glucosylation of cis-zeatin in Maize. Characterization of genes, enzymes, and endogenous cytokinins. Plant Physiol.

[83] Matsuo S, Kikuchi K, Fukuda M, Honda I, Imanishi S (2012). Roles and regulation of cytokinins in tomato fruit development. J Exp Bot.

[84] Le DT, Nishiyama R, Watanabe Y, Vankova R, Tanaka M, Seki M (2012). Identification and expression analysis of cytokinin metabolic genes in soybean under normal and drought conditions in relation to cytokinin levels. PLoS One.

[85] Davies C, Boss PK, Gerós H, Lecourieux F, Delrot S: Source/sink relationships and molecular biology of sugar accumulation in grape berries. In: The Biochemistry of the Grape Berry. Edited by Gerós H, Chaves MM, Delrot S, vol. 1: Bentham Science Publishers; 2012: 44–66.

[86] Brenner WG, Ramireddy E, Heyl A, Schmülling T (2012). Gene regulation by cytokinin in *Arabidopsis*. Frontiers in Plant Science.

[87] Thomas J, Ross CW, Chastain CJ, Koomanoff N, Hendrix JE, Van Volkenburgh E (1981). Cytokinin-induced wall extensibility in excised cotyledons of radish and cucumber. Plant Physiol.

[88] Jung KW, Seung-Ick O, Yun Young K, Kyoung Shin Y, Mei Hua C, Jeong SS (2008). Arabidopsis histidine-containing phosphotransfer factor 4 (AHP4) negatively regulates secondary wall thickening of the anther endothecium during flowering. Mol Cells.

[89] Mothes K, Engelbrecht L (1963). On the activity of a kinetin-like root factor. Life Sci.

[90] Mothes K, Engelbrecht L, Schütte HR (1961). Über die Akkumulation von α-Aminoisobuttersäure im Blattgewebe unter dem Einfluss von Kinetin. Physiol Plant.

[91] Kuiper D (1993). Sink strength: established and regulated by plant growth regulators. Plant Cell Environ.

[92] Werner T, Holst K, Pörs Y, Guivarc’h A, Mustroph A, Chriqui D (2008). Cytokinin deficiency causes distinct changes of sink and source parameters in tobacco shoots and roots. J Exp Bot.

[93] Ehness R, Roitsch T (1997). Co-ordinated induction of mRNAs for extracellular invertase and a glucose transporter in *Chenopodium rubrum* by cytokinins. Plant J.

[94] Lara MEB, Garcia M-CG, Fatima T, Ehness R, Lee TK, Proels R (2004). Extracellular invertase is an essential component of cytokinin-mediated delay of senescence. Plant Cell.

[95] Ho LC (1984). Partitioning of assimilates in fruiting tomato plants. Plant Growth Regul.

[96] Davies C, Wolf T, Robinson SP (1999). Three putative sucrose transporters are differentially expressed in grapevine tissues. Plant Sci.

[97] Zhang X-Y, Wang X-L, Wang X-F, Xia G-H, Pan Q-H, Fan R-C (2006). A shift of phloem unloading from symplasmic to apoplasmic pathway is involved in developmental onset of ripening in grape berry. Plant Physiol.

[98] Hayes MA, Davies C, Dry IB (2007). Isolation, functional characterization, and expression analysis of grapevine (*Vitis vinifera* L.) hexose transporters: differential roles in sink and source tissues. J Exp Bot.

[99] Deluc L, Grimplet J, Wheatley M, Tillett R, Quilici D, Osborne C (2007). Transcriptomic and metabolite analyses of Cabernet Sauvignon grape berry development. BMC Genomics.

[100] Nakamura N, Nakamae H, Maekawa S (1980). Effects of light and kinetin on anthocyanin accumulation in the petals of *Rosa hybrida,* Hort cv. Ehigasa. Z Pflanzenphysiol.

[101] Deikman J, Hammer PE (1995). Induction of anthocyanin acumulation by cytokinins in *Arabidopsis thaliana*. Plant Physiol.

[102] Guo J, Hu X, Duan R (2005). Interactive effects of cytokinins, light, and sucrose on the phenotypes and the syntheses of anthocyanins and lignins in cytokinin overproducing transgenic *Arabidopsis*. J Plant Growth Regul.

[103] Field SK, Smith JP, Holzapfel BP, Hardie WJ, Emery RJN (2009). Grapevine response to soil temperature: xylem cytokinins and carbohydrate reserve mobilization from budbreak to anthesis. Am J Enol Vitic.

[104] Carra A, Mica E, Gambino G, Pindo M, Moser C, Pè ME (2009). Cloning and characterization of small non-coding RNAs from grape. Plant J.

[105] Miyawaki K, Matsumoto-Kitano M, Kakimoto T (2004). Expression of cytokinin biosynthetic isopentenyltransferase genes in *Arabidopsis*: tissue specificity and regulation by auxin, cytokinin, and nitrate. Plant J.

[106] Kuroha T, Tokunaga H, Kojima M, Ueda N, Ishida T, Nagawa S (2009). Functional analyses of *LONELY GUY* cytokinin-activating enzymes reveal the importance of the direct activation pathway in *Arabidopsis*. Plant Cell.

[107] Werner T, Köllmer I, Bartrina I, Holst K, Schmülling T (2006). New insights into the biology of cytokinin degradation. Plant Biol.

[108] Pilati S, Perazzolli M, Malossini A, Cestaro A, Demattè L, Fontana P (2007). Genome-wide transcriptional analysis of grapevine berry ripening reveals a set of genes similarly modulated during three seasons and the occurrence of an oxidative burst at vèraison. BMC Genomics.

[109] Fernandes J, Tavares S, Amâncio S (2009). Identification and expression of cytokinin signaling and meristem identity genes in sulfur deficient grapevine (*Vitis vinifera* L.). Plant Signal Behav.

