# Supplementary material for: Changes in transcription of cytokinin metabolism and signalling genes in grape (Vitis vinifera L.) berries are associated with the ripening-related increase in isopentenyladenine
Source: BMC Plant Biol. 2015 Sep 16;15:223. doi: 10.1186/s12870-015-0611-5 (PMC4573921; doi:10.1186/s12870-015-0611-5)
Supplement: Additional file 1: — TAIR accession numbers of the Arabidopsis nucleotide sequences used for phylogenetic analyses. (PDF 33 kb) [file 12870_2015_611_MOESM1_ESM.pdf]

Gene name	TAIR Accession
AtIPT1	Sequence:2201294
AtIPT2	Sequence:3694256
AtIPT3	Sequence:3702189
AtIPT4	Sequence:2121981
AtIPT5	Sequence:2179631
AtIPT6	Sequence:2031207
AtIPT7	Sequence:2088027
AtIPT8	Sequence:2094130
AtIPT9	Sequence:6530300609
AtLOG1	Sequence:1005033809
AtLOG2	Sequence:1009036063
AtLOG3	Sequence:6530299313
AtLOG4	Sequence:2084053
AtLOG5	Sequence:3705872
AtLOG6	Sequence:2143475
AtLOG7	Sequence:1009065332
AtLOG8	Sequence:2143031
AtLOG9	Sequence:2180674
AtCKX1	Sequence:4515104683
AtCKX2	Sequence:3695356
AtCKX3	Sequence:2164617
AtCKX4	Sequence:1005717052
AtCKX5	Sequence:2018439
AtCKX6	Sequence:4010715489
AtCKX7	Sequence:1005717605
AtCKI1	Sequence:2061973
AtCRE1	Sequence:1005719081
AtHK1	Sequence:3696042
AtHK2	Sequence:3710958
AtHK3	Sequence:3687867
AtHK5	Sequence:4010715876
AtRR1	Sequence:1009045452
AtRR2	Sequence:5019475873
AtRR3	Sequence:2025913
AtRR4	Sequence:2194586
AtRR5	Sequence:2097872
AtRR6	Sequence:2170725
AtRR7	Sequence:2011288
AtRR8	Sequence:2040284
AtRR9	Sequence:2080592
AtRR10	Sequence:1005031868
AtRR11	Sequence:2008587
AtRR12	Sequence:5019475371
AtRR13	Sequence:2059270
AtRR14	Sequence:2065400
AtRR15	Sequence:1005036131
AtRR16	Sequence:6530299348
AtRR17	Sequence:3702839
AtRR18	Sequence:4010716191
AtRR19	Sequence:6530298673
AtRR20	Sequence:6530299927
AtRR21	Sequence:2182892
AtRR23	Sequence:2174065
