# Supplementary material for: Changes in transcription of cytokinin metabolism and signalling genes in grape (Vitis vinifera L.) berries are associated with the ripening-related increase in isopentenyladenine
Source: BMC Plant Biol. 2015 Sep 16;15:223. doi: 10.1186/s12870-015-0611-5 (PMC4573921; doi:10.1186/s12870-015-0611-5)
Supplement: Additional file 2: — Gene-specific primer pairs used for qRT-PCR analyses. (PDF 59 kb) [file 12870_2015_611_MOESM2_ESM.pdf]

Gene	NCBI Reference Sequence	Primer sequence (5'-3')	Amplicon size (bp)
VviIPT2	XM_002263711	F: CCCTCACATTCCCTTTCAGC<br>R: GGTCTACGGCAGGCTATTGG	108
VviIPT9	XM_002282976	F: TTGTGCCAGTTCTGCAAGGT<br>R: AGGCATTTCATCAACCGTGA	127
VviIPT10	XM_002279335	F: GCGAGTCGATGAAATGCTTG<br>R: ACTTTCGGAAATGCCTGTCG	140
VviIPT11	XM_002268812	F: TGGACGCCACTGAAGTGTTC<br>R: GGCGTTCCTCACCATCTCTT	152
VviIPT12	XM_002271926	F: ATGCATATGCGCAAGCAAAC<br>R: CCCATTACCACCACCACCTT	110
VviIPT13	XM_003632592	F: AGGGGTGGGCTAGTGGAAGA<br>R: GCCCTCAAGTAGGCGTCCAT	110
VviIPT14	XM_002277555	F: AAGCACAGACGCCCCTACTG<br>R: CCGGTGGCTCCTATCACGAA	100
VviIPT15	XM_002278900	F: CATACATGTCTCGATCCCACCG<br>R: ACCAAAACCTGACCATGGTTGC	95
VviCYP735A	XM_002280169	F: GGCAGCTCTCGTCTCCAAAT<br>R: GGCAGCTCTCGTCTCCAAAT	112
VviLOG5a	XM_010665788	F: CAAGCTGGGCCCATGTGATG<br>R: GGAGAGGTTCGACCCGTAGC	136
VviLOG5b	XM_002281803	F: TCAAGCCTTCACAGCGCCAT<br>R: CCTCGCCTTTGCAATGACCC	110
VviLOG10	XM_002276739	F: CACATGGGCTCAGCTGGGTA<br>R: TGACGGGCAGCTGCTGTTAT	134
VviLOG11	XM_002275378	F: GTCCGCACCAACAGCCAAAG<br>R: GCCACACCAGGCTTCTCTGA	129
VviLOG12	XM_002276243	F: TCATTAGTCCCAGTGCCCGC<br>R: GCTGCTCCATCTCCCAGCTC	129
VviLOG13	XM_002285210	F: GGCAGTGGGGGCTGATACAA<br>R: TCCATGACAAACCGGTGGGA	132
VviLOG14	XM_002285680	F: TATCAGGGCATGCGGTTGGG<br>R: TCCATACCCACCAGGAAGTGC	110
VviLOG15	XM_002274711	F: TTTCAGCAACCACGGCAGAAG<br>R: TGGTCCACTTCCCAGGTTTG	97
VviLOG16	XM_002277816	F: CAGTGGGGCTATTGAATGTGGAC<br>R: AGTTCTTCTGCCGTGTCTGCTG	130
VviLOG17	XM_002278269	F: AGCATGTGGGCTTCATGTCTT<br>R: CCAGTTGCGTGAAAGAGCGT	111
VviCKX5	XM_002280761	F: TCTCAGCAATCAGAACCGTCAGA<br>R: GGGGTCGAAGTCCATCTTTC	160
VviCKX6a	XM_002270805	F: TTGTGGGAGGTTCCACACCC<br>R: GAATGGGGCCATTGCTGGTG	115
VviCKX6b	XM_002284524	F: TGCGAGAATCCTGCTCCATGA<br>R: AAGTGGCAAGCGCACTTTGG	138
VviCKX7	XM_002279924	F: TCCTCAAGCTAGGGTTGCCG<br>R: GGAAGGGTGGAAGCGACACT	132
VviCKX8	XM_002279483	F: CATGGGCTGATGTCCTCCCC<br>R: GCAGCTGGGGTTTGTTGGAC	127
VviCKX9	XM_003632356	F: TCCTGCACAATTGTCCTCCTAACA<br>R: TCTGAGACTTCATCAACTGTATGGC	160
VviCKX10	XM_002263610	F1: TTGCACCAGTGTCGTGGACT<br>R1: TGATCTGAGGACCATGGCGG<br>F2: ACTTCTCCACATTTTCTAGAGACCA<br>R2: ATTAGGAGGACTATTTTGCATGGAG	no product
VviCKX11	XM_002264409	F: TCGCTCTAAAACCGGCACCA<br>R: TGCATCAAGAGTGAACCTTCCA	136
VviCHK2	XM_002269941	F: ATTGTCACCTGCGTGGACAG<br>R: CCCATCCATCTCTGGCATTT	105
VviCHK3	XM_002276925	F: GGAGCTGATGTCGTCTGTGC<br>R: GCTTCAAACCCGTCCATTTC	119
VviCHK4	XM_002285081	F: AGCCATCCATCCCACATTTC<br>R: ACGGCTGCTCGAGAGTAAGG	158

Gene	NCBI Reference Sequence	Primer sequence (5’-3’)	Amplicon size (bp)
VviRR11a	XM_002274637	F: TTGAGAGCGCATAGGCGTGA	128
		R: TGCAGTCTGCTCCACAGCAT	
VviRR11b	XM_002267580	F: TGCCAAATGGTAAACCCTCAAGC	140
		R: AGAGCTGACCCACTCCCTGA	
VviRR25	XM_002269335	F: TTGGCCAGGGAATGGTCTCC	131
		R: GCCCGGCCTAGGCATACTTT	
VviRR26	XM_002270082	F: AGGATGCATTTGCCTGGGCT	95
		R: ACCTTTGGCCCACTGAACTGT	
VviRR27	XM_002275106	F: AGCTGCATGATATGCGAACGTG	129
		R: TGCCCCATACCCAACACAACT	
VviRR28	XM_002281255	F: ACCCAAGCACTGTCCAGAGA	120
		R: GGCGCAAATTCTCAGTGCCT	
VviRR29	XM_002270797	F: TGGGCGTCAGTGGTTCATCT	114
		R: ACGGATCGCGATTGAGTGGT	
VviRR30	XM_002282892	F: GGCGTCTTGTCCTGTTCCTG	137
		R: GTTGAAGCAAGGTTGCCCCT	
VviRR31	FJ822980 (partial cds)	F: CCGGGAAACTCCGGTGGTAA	128
		R: CTTTCAGCCGCTTCACGTCC	
VviRR32	XM_002283751	F: CATACCCTGTCATCCCAGGCA	133
		R: ACGGCTGTGGAGAGTGGAAC	
VviRR33	XM_002280710	F: TCCTGCCCCTAGGGATGGAT	149
		R: AAGTGCCTGGAGGAAGGAGC	
VviRR34	XM_002284468	F: CCCGGGATGAGCGGTTATGA	126
		R: TGCCCCTCCTTCCAAACACA	
VviRR35	XM_002273954	F: GCTAGCTGTTGATGACAGCCTT	147
		R: ACGGAAGGTGTGTTTGGGTCA	
VviRR36	XM_002266214	F1: CCAGTTGTGATCATGTCCTCTGA	no product
		R1: GCGAGACCTCAACATCTACGAGT	
		F2: AATCATCACCAAGGAACGAAAGT	
		R2: TGATCACAACTGGGATGCCT	
VviRR37	XM_002268316	F: AGCTTCAAAGGCTGTTCATGTCT	116
		R: TCGATTGAAGACATTTTTGGAGGGT	
VviRR38	XM_002267339	F: AGCTGTTGATGACAGCCTCGT	124
		R: TGCCTCTTGCAAACCCAGGA	
VviRR39	XM_002267896	F: TGGGTATTGCTGCAGAGACACAG	129
		R: CCTTACTCCCAGAATCCACAGCA	
VviRR40	XM_003634849	F: TCTAGCTGTTGATGACAGCCTCATT	138
		R: CTTTCCTGTTCTGCCTCTTGCAC	
VviRR41	XM_002267368	F: TGTTTGGAAGAAGGAGCAGAA	91
		R: CTTTTCCCTTCAGTAGATGGGG	
