# Supplementary figures and images for: Changes in transcription of cytokinin metabolism and signalling genes in grape (Vitis vinifera L.) berries are associated with the ripening-related increase in isopentenyladenine
Source: BMC Plant Biol. 2015 Sep 16;15:223. doi: 10.1186/s12870-015-0611-5 (PMC4573921; doi:10.1186/s12870-015-0611-5)

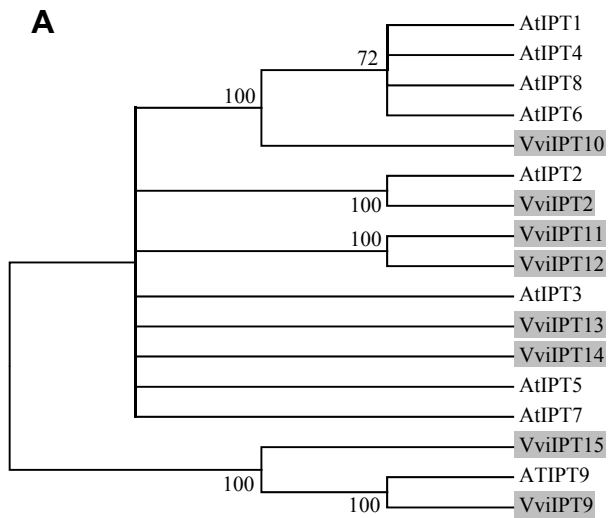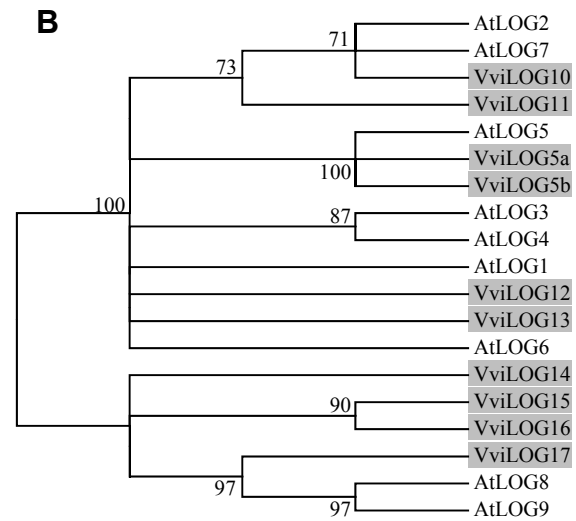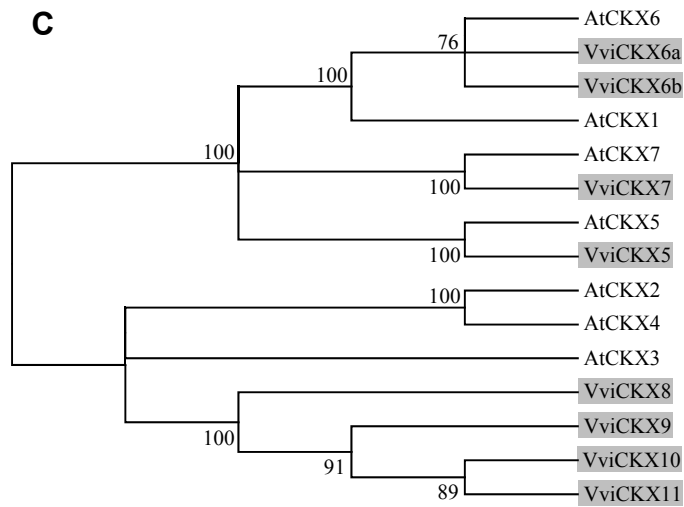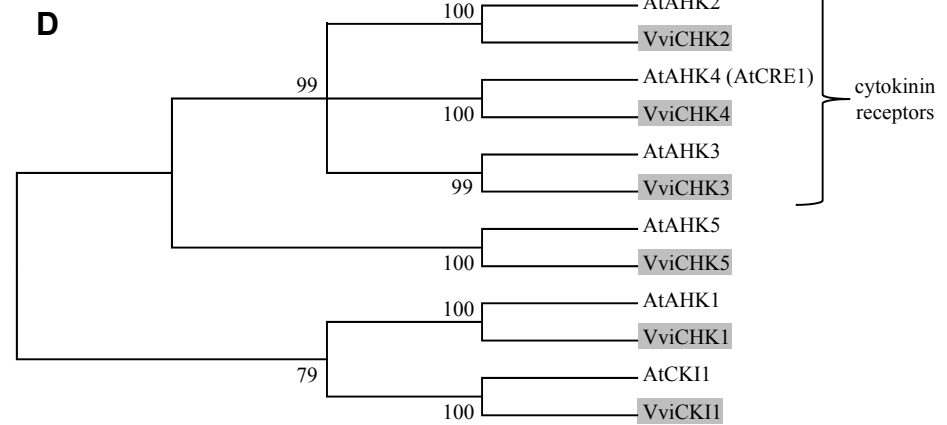

Supplement: Additional file 3: — Phylogenetic relationship of IPT , LOG , CKX and CHK coding sequences from grapevine and Arabidopsis. Unrooted trees of (A) IPT, (B) LOG, (C) CKX and (D) CHK sequences were generated from alignments created with MUSCLE [45], all positions containing gaps and missing data were eliminated. The evolutionary history was inferred by using the Maximum Likelihood method based on the JTT matrix-based model [46]. A bootstrap consensus tree was generated from 100 replicates [47] and branches corresponding to partitions replicated in less than 70 % replicates were collapsed. Initial tree(s) for the heuristic search were obtained automatically by applying Neighbor-Join and BioNJ algorithms to a matrix of pairwise distances estimated using a JTT model and then selecting the topology with superior log value. The coding data was translated assuming a standard genetic code table. The naming of grapevine genes followed the guidelines published by Grimplet et al. [48]. Grapevine sequences are highlighted with a grey background. NCBI or TAIR accession numbers for all sequences used in the phylogenetic analysis are listed in Table 2 and Additional file 1. (PDF 52 kb) [file 12870_2015_611_MOESM3_ESM.pdf]

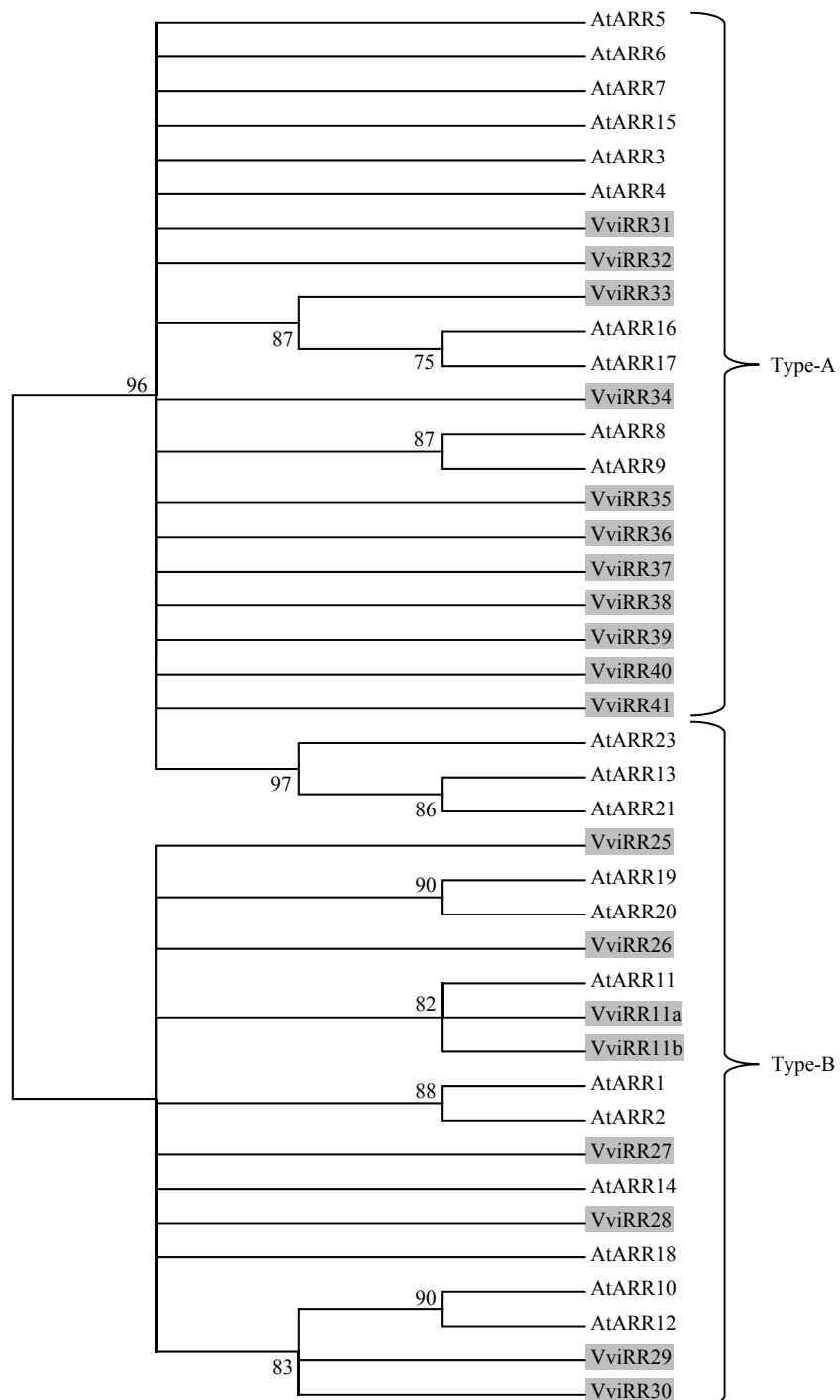

Supplement: Additional file 4: — Phylogenetic relationship of RR coding sequences from grapevine and Arabidopsis. The unrooted tree was generated from an alignment created with MUSCLE [45], all positions containing gaps and missing data were eliminated. The evolutionary history was inferred by using the Maximum Likelihood method based on the JTT matrix-based model [46]. A bootstrap consensus tree was generated from 100 replicates [47] and branches corresponding to partitions replicated in less than 70 % replicates were collapsed. Initial tree(s) for the heuristic search were obtained automatically by applying Neighbor-Join and BioNJ algorithms to a matrix of pairwise distances estimated using a JTT model and then selecting the topology with superior log value. The coding data was translated assuming a standard genetic code table. The naming of grapevine genes followed the guidelines published by Grimplet et al. [48]. Grapevine sequences are highlighted with a grey background. NCBI or TAIR accession numbers for all sequences used in the phylogenetic analysis are listed in Table 2 and Additional file 1. (PDF 31 kb) [file 12870_2015_611_MOESM4_ESM.pdf]

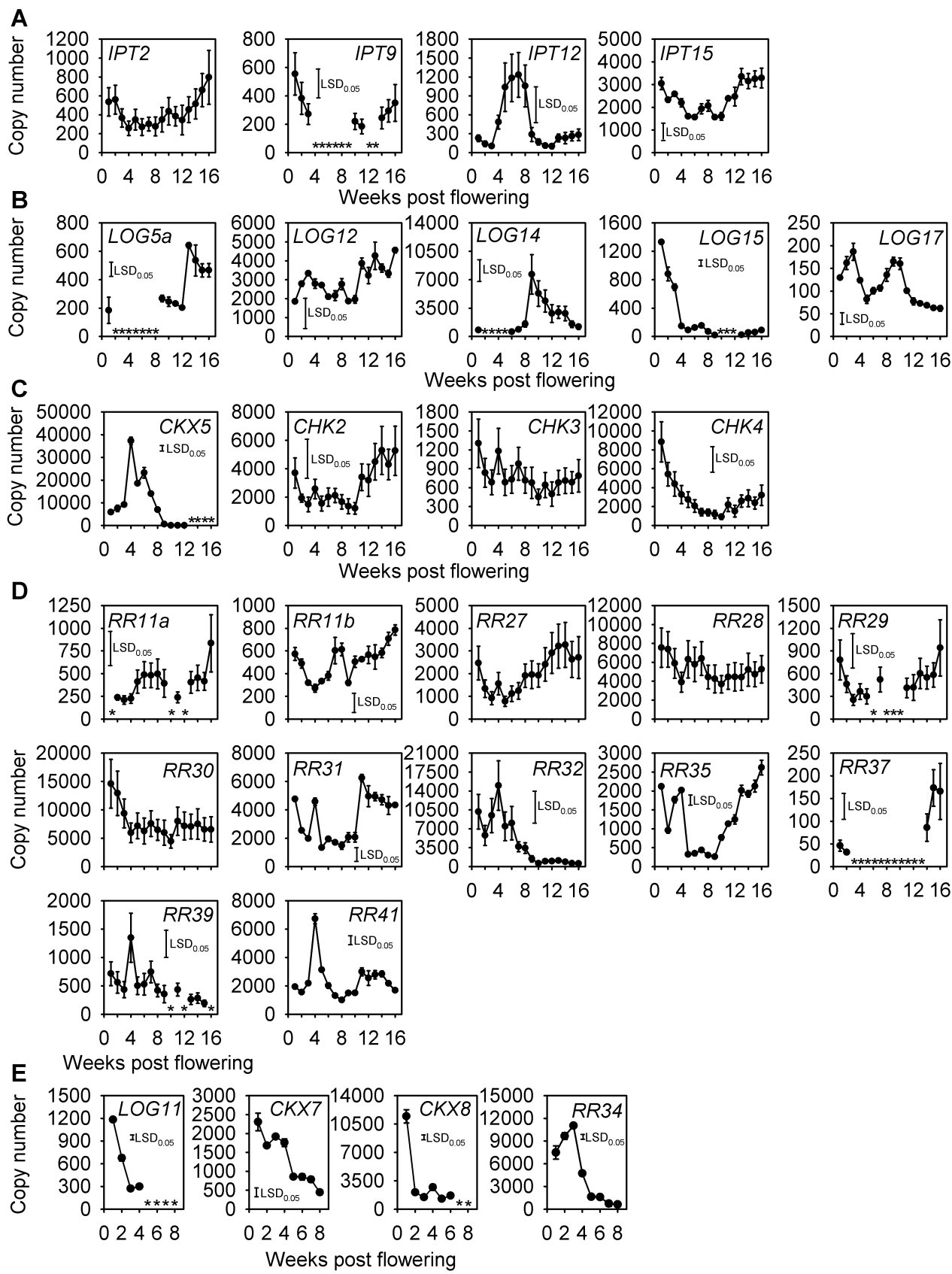

Supplement: Additional file 5: — Transcript accumulation of cytokinin-related genes expressed at two or more time points in a Shiraz berry developmental series. The expression of (A) IPT, (B) LOG, (C) CKX, (D) RR and (E) pre-veraison-specific genes was analysed by qRT-PCR. All data represent means (n = 3) ± SE and LSD values were determined at the p <0.05 significance level. Asterisks mark samples in which expression could not be detected. (PDF 188 kb) [file 12870_2015_611_MOESM5_ESM.pdf]

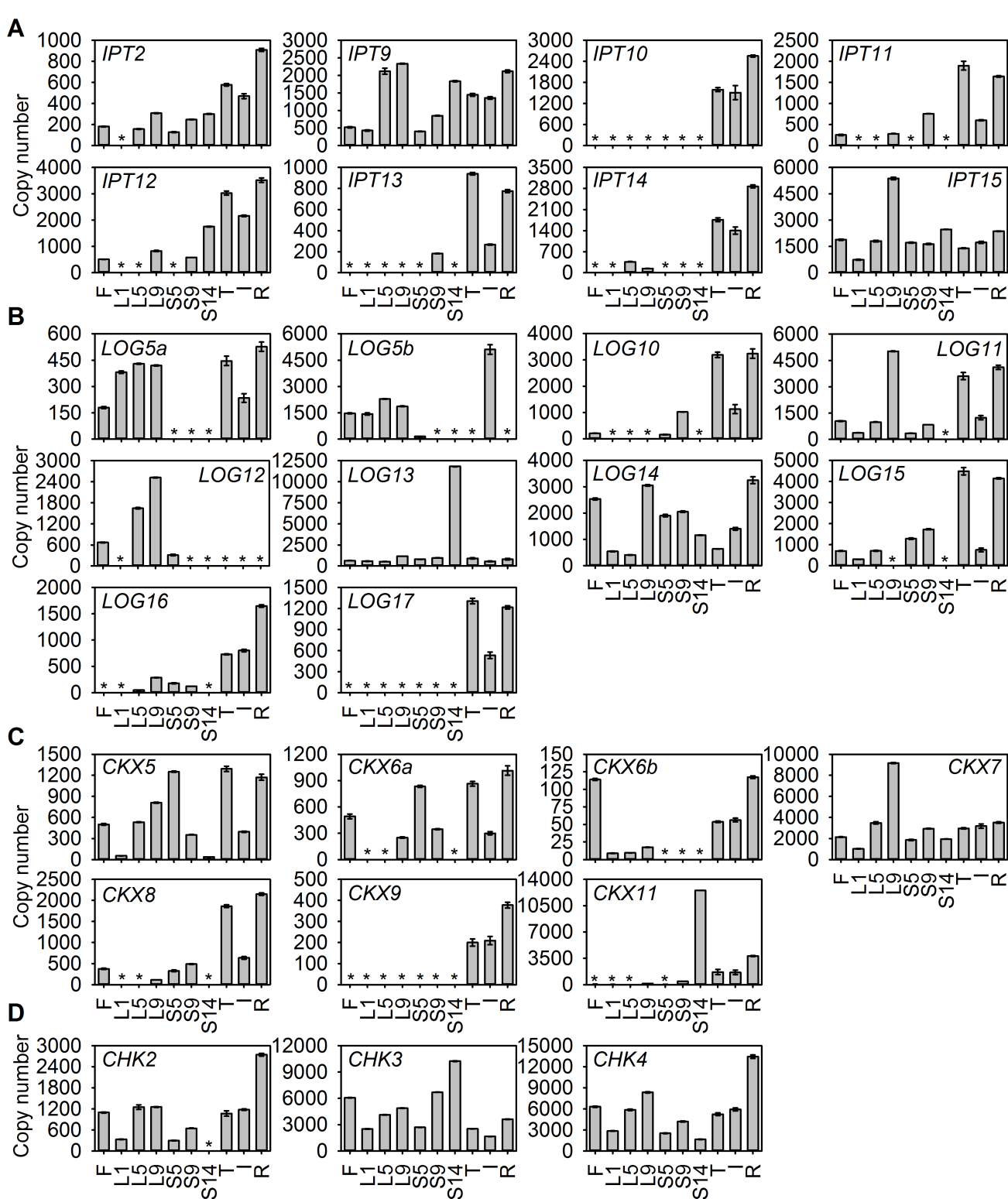

Supplement: Additional file 6: — Transcript accumulation of IPT , LOG , CKX and CHK genes in different Shiraz tissues. The expression of (A) IPT, (B) LOG, (C) CKX and (D) CHK genes was analysed by qRT-PCR. All data represent means ± SE (n = 3 technical replicates). Asterisks mark tissues in which expression could not be detected. F, flower; I, internode; L, leaf (node indicated by number); R, root; S, seed (wpf indicated by number); T, tendril. (PDF 493 kb) [file 12870_2015_611_MOESM6_ESM.pdf]

Copy number

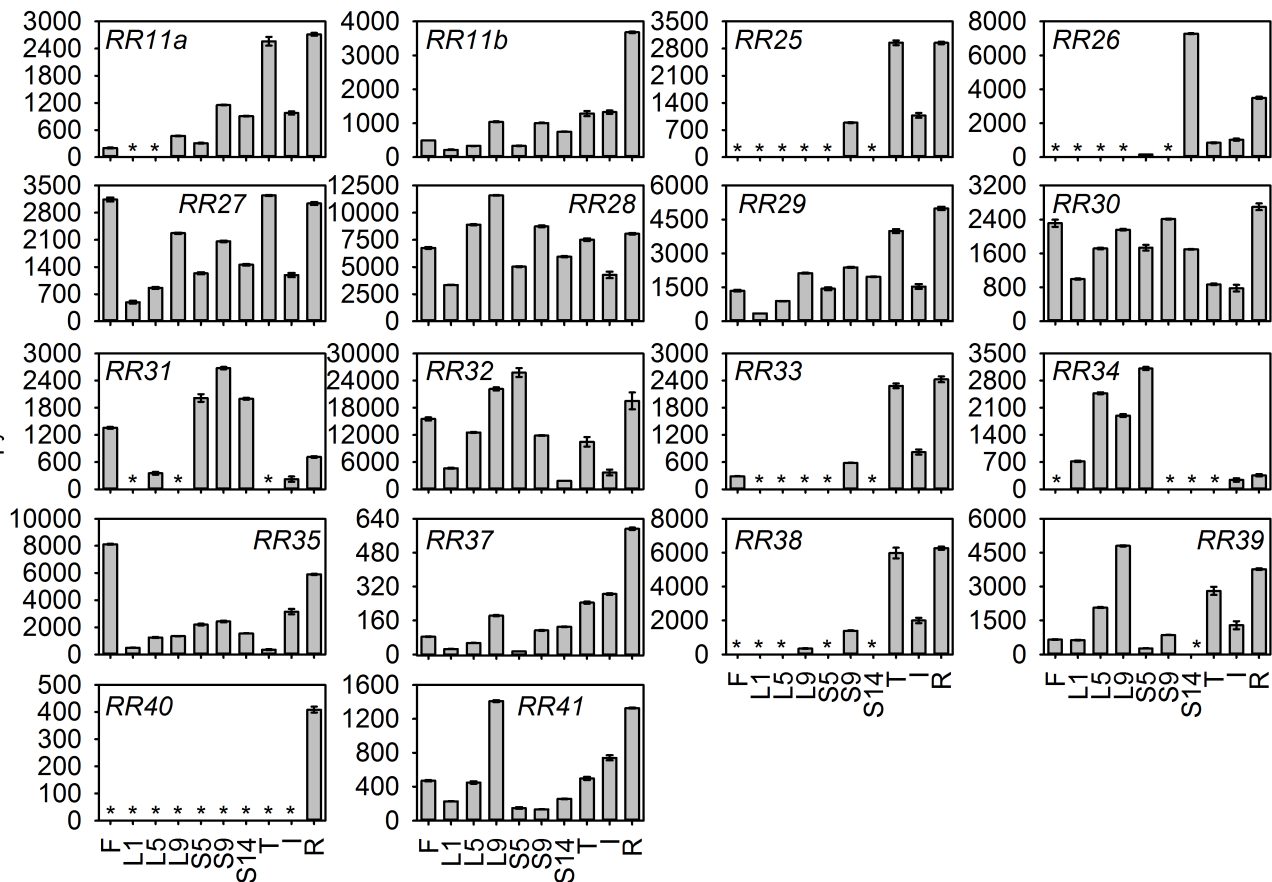

Supplement: Additional file 7: — Transcript accumulation of RR genes in different Shiraz tissues. The expression of RR genes was analysed by qRT-PCR. All data represent means ± SE (n = 3 technical replicates). Asterisks mark tissues in which expression could not be detected. F, flower; I, internode; L, leaf (node indicated by number); R, root; S, seed (wpf indicated by number); T, tendril. (PDF 310 kb) [file 12870_2015_611_MOESM7_ESM.pdf]
